# Exosome markers associated with immune activation and oxidative stress in HIV patients on antiretroviral therapy

**DOI:** 10.1038/s41598-018-25515-4

**Published:** 2018-05-08

**Authors:** Sukrutha Chettimada, David R. Lorenz, Vikas Misra, Simon T. Dillon, R. Keith Reeves, Cordelia Manickam, Susan Morgello, Gregory D. Kirk, Shruti H. Mehta, Dana Gabuzda

**Affiliations:** 1 Department of Cancer Immunology and Virology, Dana-Farber Cancer Institute, Boston, MA USA; 2 BIDMC Genomics, Proteomics, Bioinformatics and Systems Biology Center, Beth Israel Deaconess Medical Center, Boston, MA USA; 3 Department of Medicine, Beth Israel Deaconess Medical Center, Harvard Medical School, Boston, MA USA; 4 Center for Virology and Vaccine Research, Beth Israel Deaconess Medical Center, Boston, MA USA; 5 Department of Neurology, Neuroscience and Pathology, Mount Sinai Medical Center, New York, NY USA; 6 Department of Epidemiology, Bloomberg School of Public Health, Johns Hopkins University, Baltimore, MD USA; 7 Department of Neurology, Harvard Medical School, Boston, MA USA

## Abstract

Exosomes are nanovesicles released from most cell types including immune cells. Prior studies suggest exosomes play a role in HIV pathogenesis, but little is known about exosome cargo in relation to immune responses and oxidative stress. Here, we characterize plasma exosomes in HIV patients and their relationship to immunological and oxidative stress markers. Plasma exosome fractions were isolated from HIV-positive subjects on ART with suppressed viral load and HIV-negative controls. Exosomes were characterized by electron microscopy, nanoparticle tracking, immunoblotting, and LC-MS/MS proteomics. Plasma exosomes were increased in HIV-positive subjects compared to controls, and correlated with increased oxidative stress markers (cystine, oxidized cys-gly) and decreased PUFA (DHA, EPA, DPA). Untargeted proteomics detected markers of exosomes (CD9, CD63, CD81), immune activation (CD14, CRP, HLA-A, HLA-B), oxidative stress (CAT, PRDX1, PRDX2, TXN), and Notch4 in plasma exosomes. Exosomal Notch4 was increased in HIV-positive subjects versus controls and correlated with immune activation markers. Treatment of THP-1 monocytic cells with patient-derived exosomes induced expression of genes related to interferon responses and immune activation. These results suggest that exosomes in ART-treated HIV patients carry proteins related to immune activation and oxidative stress, have immunomodulatory effects on myeloid cells, and may have pro-inflammatory and redox effects during pathogenesis.

## Introduction

Chronic inflammation is a hallmark of HIV infection associated with disease progression and comorbidities. In antiretroviral therapy (ART)-treated HIV patients, chronic inflammation can be caused by multiple factors, including ongoing HIV replication, increased type-I and II interferons, microbial translocation, dysregulated chemokine and cytokine production, substance use, and co-infections^1–3^. Current ART regimens suppress HIV replication, but low CD4 T cell counts, immune activation, and chronic inflammation persist, frequently associated with oxidative stress and elevated rates of comorbidities. Identifying biomarkers related to chronic inflammation, oxidative stress, and their underlying causes is important to monitor disease progression and therapeutic responses, and elucidate mechanisms of pathophysiology

Exosomes carry specific protein and nucleic acid cargo that can serve as biomarkers of many diseases including HIV, cancer, and neurodegenerative diseases^4–10^. Furthermore, the cargo-carrying capacity of exosomes is being leveraged to deliver therapeutic agents^5^. Exosomes are 30–150 nm extracellular vesicles (EVs) secreted into the extracellular milieu by most cell types. They have been detected in body fluids such as blood, urine, and semen, as well as extracellular matrix in tissues, and carry cargo involved in mediating communication between cells and organs. Exosome protein and nucleic acid cargo is influenced by cell type of origin, and biological processes including immune activation, inflammation, and exposure to stressors^6,11–13^. Exosomes have physiological functions that contribute to maintaining human health, but have also been implicated in contributing to pathophysiology of many diseases^5,13–16^.

HIV and other retroviruses exploit pre-existing cellular pathways for vesicle trafficking, assembly, and release for viral dissemination and pathogenesis^9,10,17–20^. Furthermore, HIV infection induces exosome release from monocytes^21^, macrophages^20^, dendritic cells^22^, and T cells^16^. Exosomes secreted from HIV-infected cells transport viral and host components that can facilitate viral dissemination through body fluids^19,22–27^. Most studies on exosomes in HIV infection have focused on detrimental effects, including pro-inflammatory or pro-apoptotic effects^8,16,18,22,23,25,26^. For example, HIV Nef protein was shown to induce release of Nef-positive exosomes from T cells, leading to bystander apoptosis *in vitro*^16^. However, exosomes can also play beneficial roles. For example, exosomes can promote antiviral activity by cell-to-cell transfer of innate antiviral factors such as APOBEC3G, and cytokines that inhibit viral replication such as type I interferons^28,29^. Exosomes may also serve protective functions during oxidative stress^30^.

A previous study showed that HIV-infected individuals have more abundant plasma exosomes compared to healthy controls^7^. HIV infection also influences the size and cargo of other plasma EVs^7,8^. However, little is known about the relationship of circulating exosome cargo to immune responses, and how these processes are altered by HIV infection. Here, we perform a cross-sectional study to characterize the protein cargo of circulating exosomes in a cohort of HIV-positive subjects on ART and uninfected HIV-negative controls, and determine the relationship of exosome protein cargo to virological, immunological, and oxidative stress markers. We then examine transcriptional changes induced in recipient monocytic cells following uptake of patient-derived exosomes to evaluate their functional effects on immune activation and inflammation.

## Results

### Characteristics of the study cohort

The study cohort consisted of 43 HIV-positive and 34 HIV-negative subjects. Demographic and clinical characteristics are summarized in Table 1. HIV-positive subjects were predominantly male (70%), 39.5% white, with mean age 48 years (range, 35–65 years). All HIV-positive subjects were on ART with suppressed or low viral load (VL) (<400 or <2500 HIV RNA copies/ml, respectively) and mean CD4 count 348 cells/μl. Subjects with undetectable plasma VL (<50 or <400 HIV RNA copies/ml depending on sensitivity of assay used at the time) were classified as aviremic (53.5%), and those with detectable VL between 400–2500 HIV RNA copies/ml as viremic (46.5%). Among HIV-positive subjects, 70% were smokers, 49% reported recent cocaine use, 44% had current CD4 counts <300 cells/μl, 72% had nadir CD4 counts below 300 cells/μl, and 42% had HCV co-infection. Healthy control subjects were HIV- negative individuals matched for age, race, gender, and smoking.

**Table 1:** Demographic and clinical characteristics of the study cohort.

Characteristic		HIV-negative (n = 34)	HIV-positive (n = 43)
Years of infection^α^		0	14 (9.5–21.0)
ART use duration (months)^α^		0	29.1 (11.7–75.1)
Age (years)^α^		53.5 (48.2–56.7)	48.1 (43.4–51.0)
Gender (% male)		64.7	69.7
Race (%)	White	35	39.5
	Non-white	65	60.5
CD4 count (cells/µl)^α^		—	342 (195–461.5)
Nadir CD4 (cells/µl)^α^		—	83.5 (28.7–288.5)
CD8 count (cells/µl)^α^		—	836.5 (719–1357)
CD4/CD8 ratio^α^		—	0.25 (0.12–0.46)
Plasma viral load (HIV RNA copies/ml)^α^		—	404 (40–926.5)
Viral load <400 HIV RNA copies/ml^γ^ (%)		—	53.5
HCV seropositive (%)		—	42
Cocaine use (%)^β^		—	49
Alcohol use (%)^β^		—	40
Smoking (%)		79	70

^α^Median (Interquartile range).

^β^Based on patient report.

^γ^Viral load below limit of detection.

ART = Antiretroviral therapy, HCV = hepatitis C virus, –Not available.

### Characterization of plasma exosome fractions

**Figure 1:**
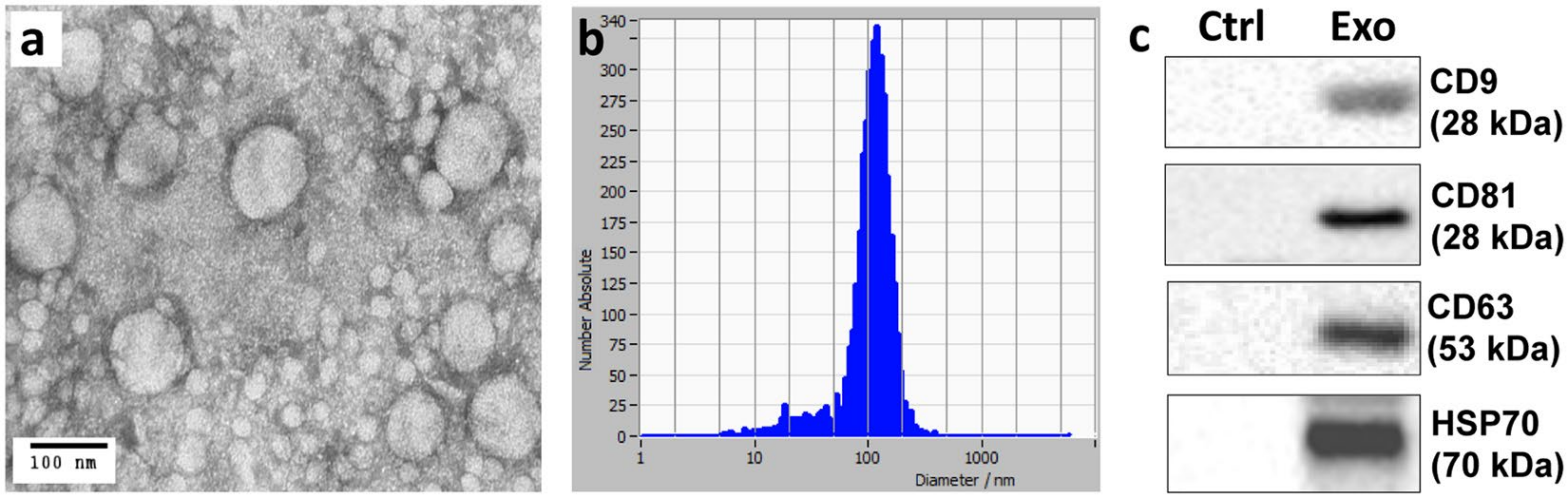
Characterization of exosome fractions isolated from plasma. Morphological characterization of plasma exosome fractions by transmission electron microscopy **(a)** and vesicle size distribution measured by nanoparticle tracking analysis (NTA) **(b)** from one representative HIV-negative subject. **(c)** Detection of exosome markers CD9, CD81, CD63, and HSP70 in plasma exosome fractions by immunoblotting (25 μg protein/lane) in control (Ctrl) (no exosome fraction) and plasma exosome fraction (Exo).

**Figure 2:**
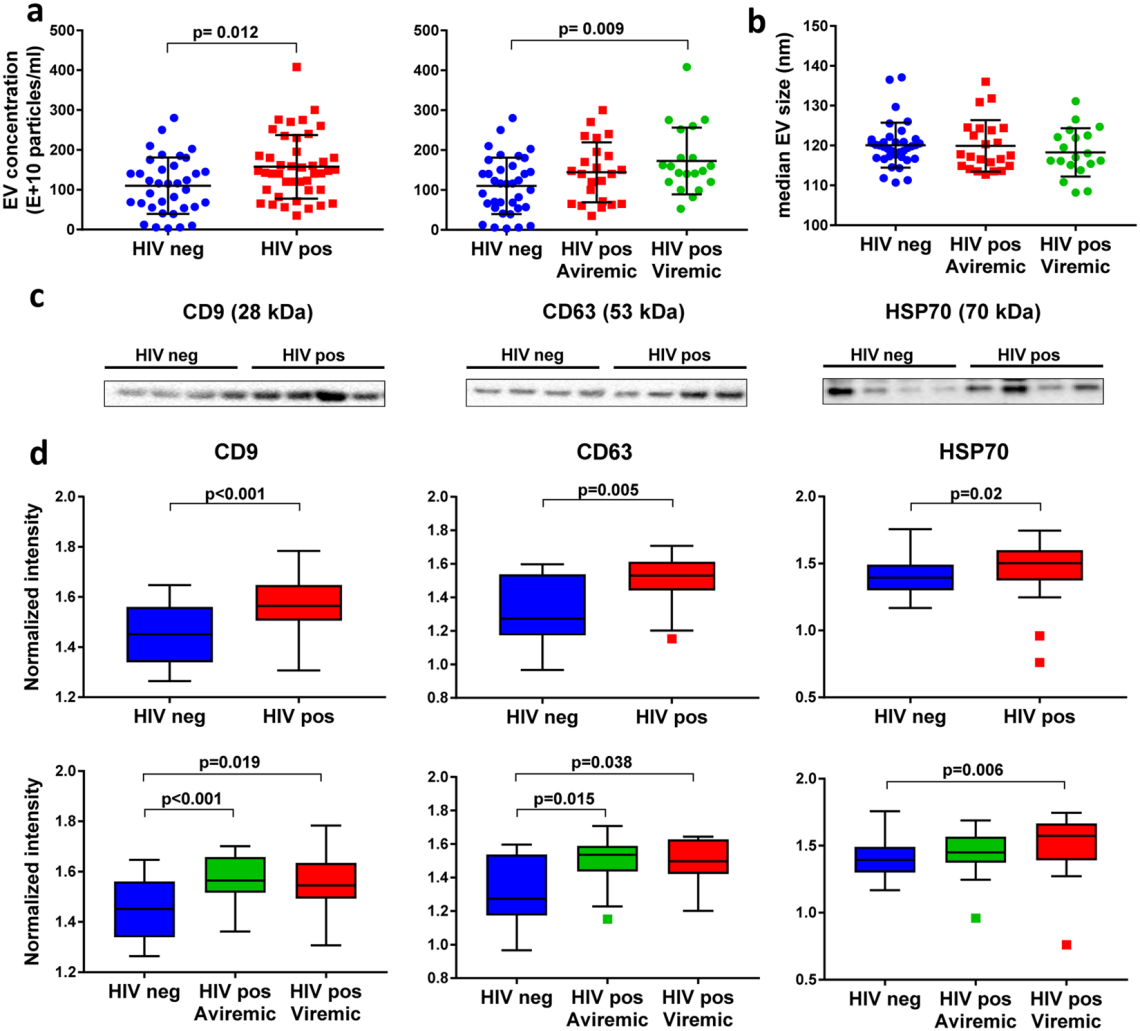
Plasma exosomes and exosome markers CD9, CD63, and HSP70 are elevated in viremic HIV-positive subjects on ART compared to controls. Exosome fractions were isolated from plasma (0.4 ml) of HIV-positive subjects (aviremic and viremic with viral load <400 and 400–2500 HIV RNA copies/ml, respectively) and HIV-negative controls. Beeswarm plots show EV concentration (**a**) and median size (**b**) measured by NTA in aviremic (n = 23) and viremic (n = 20) HIV-positive versus HIV-negative subjects (n = 34). Horizontal bars represent means and error bars represent SD. (**c**) Proteins (25 μg protein per lane) were separated by SDS-PAGE and immunoblotted with exosome marker antibodies against CD9, CD63, and HSP70. Representative blots from 4 HIV-negative and 4 HIV-positive samples are shown. Bands in each lane were normalized to corresponding EV numbers (full length blots for all samples are shown in Supplementary Figure S3). (**d**) Box plots in upper panel show exosome marker protein levels in HIV-positive (n = 37) versus HIV-negative subjects (n = 27). Box plots in lower panel show exosome marker protein levels in aviremic (n = 21) and viremic (n = 16) HIV-positive versus HIV-negative subjects (n = 27). Medians are represented by horizontal bars, boxes span the interquartile range (IQR), and whiskers extend to extreme data points within 1.5 times IQR. Outliers are plotted outside 1.5 times the IQR. p-values were calculated by Mann-Whitney U test.

To characterize circulating exosomes in HIV and control subjects, plasma from aviremic and viremic HIV-positive subjects on ART (n = 43) and HIV-negative controls (n = 34) was treated with ExoQuick precipitating reagent. Isolated exosome fractions were filtered (0.22 μm) to eliminate larger co-isolated vesicles^31^. To validate results from the ExoQuick method, exosome fractions were also isolated from pooled plasma of HIV-positive and HIV-negative subjects by differential ultracentrifugation (Supplementary Figure S1). Exosome fractions were examined for morphological and molecular characteristics. Transmission electron microscopy (TEM) revealed vesicles of 50–100 nm in diameter, in addition to particles of smaller sizes (Fig. 1a). Nanoparticle tracking analysis (NTA) yielded histograms showing that the majority of particles were 50–150 nm diameter, with a peak at 100–120 nm and concentrations in the range of 10^11^–10^12^ per ml in plasma (Figs 1b and 2a). Exosome isolation by differential ultracentrifugation of pooled plasma resulted in exosome fractions with higher purity, but lower yield, compared to the ExoQuick method based on TEM, dynamic light scattering (DLS), and NTA studies of isolated exosome fractions (Supplementary Figure S1). Immunoblotting detected the exosome markers CD9, CD81, CD63, and HSP70 in plasma exosome fractions (Fig. 1c). The ER membrane marker calnexin was absent in plasma exosome preparations (Supplementary Figure S2), suggesting exosome fractions were free of ER membrane contamination^32^.

### Plasma exosomes and exosome markers are elevated in viremic HIV-positive subjects on ART compared to controls

Based on NTA measurements, EV numbers were more abundant in HIV-positive (n = 43) compared to control subjects (n = 34) (2.95 vs. 1.4 × 10^11^ particles/ml, respectively, in the 30–150 nm size range; p = 0.012) (Fig. 2a left panel), with higher EV numbers detected by NTA in viremic (n = 23) but not aviremic (n = 20) HIV-positive compared to control subjects (p = 0.009) (Fig. 2a right panel). However, there was no significant difference in median EV size between the three groups (p > 0.20) (Fig. 2b). To determine whether exosomes are likely to account for the increased abundance of EV detected in HIV-positive subjects, we compared plasma exosome marker levels in HIV-positive subjects (n = 37) to those of HIV-negative subjects (n = 27) by immunoblotting (Fig. 2c). Exosome markers CD9, CD63, and HSP70 were detected at higher levels in plasma exosome fractions from HIV-positive compared to HIV-negative subjects (Fig. 2d upper panel, p < 0.001, p = 0.005, p = 0.02 for CD9, CD63, and HSP70, respectively). Exosome fractions isolated by differential ultracentrifugation showed increased exosome marker levels (CD9, CD63, HSP70) in pooled HIV-positive compared to HIV-negative plasma, validating results from the ExoQuick method (Supplementary Figure 1c). To test whether this increase in exosome marker levels was due to increased EV loading of HIV-positive samples, we loaded equal EV numbers on gels. Exosome marker levels (CD9, CD63, Flotillin-1, and HSP70) were higher in HIV-positive than HIV-negative samples when equal EV numbers were loaded, suggesting increased exosomes in HIV-positive plasma EV fractions (Supplementary Figure 1c). Levels of these exosome markers were higher in plasma exosome fractions from viremic HIV-positive compared to HIV-negative subjects (Fig. 2d, lower panel, p < 0.05); CD9 (p < 0.001) and CD63 (p = 0.015), but not HSP70 (p = 0.18), were also higher in plasma exosome fractions from aviremic HIV-positive compared to HIV-negative subjects. These results suggest that plasma exosomes contribute to increased EV numbers in HIV-positive subjects. Given greater abundance of plasma exosome markers in HIV-positive vs. control subjects, we examined relationships between these markers and virological or immunological parameters in HIV-positive subjects by correlation analyses of EV numbers or exosome marker levels (CD9, CD63, HSP70), plasma VL, CD4 counts, and CD4:CD8 ratios. No significant correlations were observed between EV numbers or exosome marker levels in HIV-positive subjects and these markers of HIV disease progression (p > 0.05). These results suggest that EV numbers and exosome marker levels are elevated in HIV-positive subjects on ART with viral suppression or low-level viremia as compared to HIV-negative controls, but do not show significant correlations with plasma VL, CD4 cell counts, or CD4:CD8 ratios in these patients.

### Exosome markers correlate with increased oxidative stress markers and reduced n-3 and n-6 PUFA

**Figure 3:**
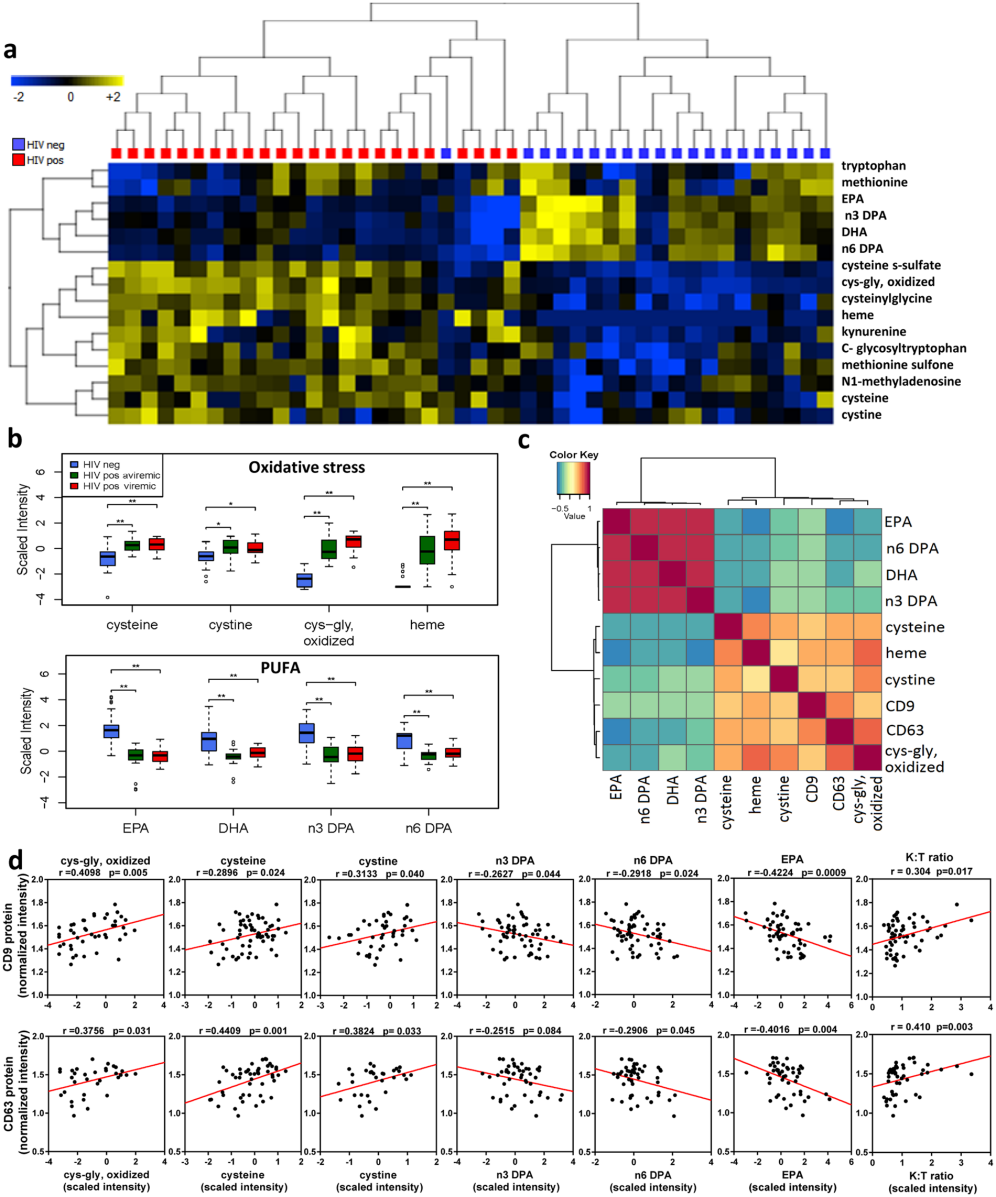
Exosome markers correlate positively with oxidative stress-related metabolites and negatively with levels of n-3 and n-6 polyunsaturated fatty acids. (**a**) Heatmap shows unsupervised hierarchical clustering of metabolites (n = 16) associated with oxidative stress, tryptophan catabolism, and polyunsaturated fatty acid (PUFA) metabolism that distinguish HIV-positive from HIV-negative control subjects (FC > 1.3, p < 0.05, FDR < 0.10). (**b**) Metabolites associated with oxidative stress are increased, and the indicated PUFA are decreased in aviremic and viremic HIV-positive subjects versus controls (*p < 0.01, **p < 0.001). Medians represented by horizontal bars, boxes span the IQR, and whiskers extend to extreme data points within 1.5 times IQR. Outliers are plotted outside 1.5 times the IQR. P-values calculated by Welch’s t-test (p < 0.05; n = 26 HIV-negative, n = 21 HIV-positive aviremic, n = 16 HIV-positive viremic subjects). (**c**) Pearson correlation matrix r-values show positive correlation of exosome marker proteins (CD9 and CD63) with metabolites associated with oxidative stress, and negative correlation with n-3 and n-6 PUFA (p < 0.05). (**d**) Correlation scatter plots are shown with correlation coefficients and p-values above each plot. n = 36 HIV-positive, 26 HIV-negative. EPA, eicosapentaenoate (20:5n3); DHA, docosahexaenoate (22:6n3); n3 DPA, docosapentaenoate (22:5n3); n6 DPA, docosapentaenoate (22:5n6); K:T ratio, kynurenine: tryptophan ratio. Scatter plots showing Pearson correlation plots for additional exosome markers and metabolites are in Supplementary Figure S4.

**Figure 4:**
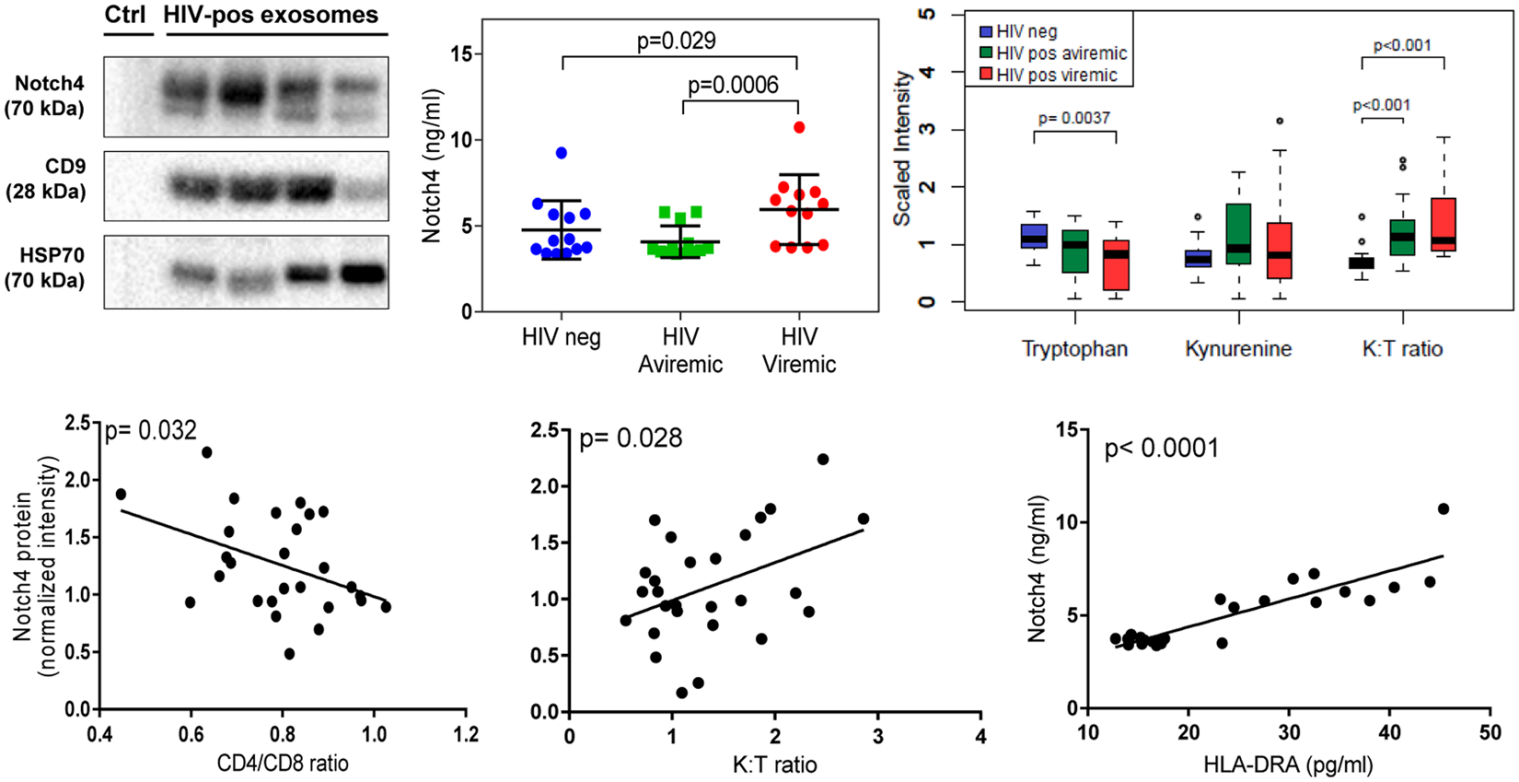
Notch4 protein is detected in plasma exosome fractions from HIV-positive subjects on ART and correlates with immune activation markers. Plasma exosome fraction (25 μg protein per lane, Ctrl = PBS blank) immunoblotted with Notch4 and exosome marker antibodies (top left). Blots are cropped and full-length blots are included in Supplementary Figure S6. Notch4 protein measured by ELISA in exosome fractions from aviremic and viremic HIV-positive versus HIV-negative subjects (top middle). Horizontal bars represent mean, error bars represent SD. P-values calculated by Mann-Whitney U test. Plasma K:T ratio is increased in aviremic and viremic HIV-positive subjects versus controls (top right). Medians in boxplots are represented by horizontal bars, boxes span the interquartile range (IQR), and whiskers extend to extreme data points within 1.5 times IQR. Outliers are plotted outside 1.5 times IQR. P-values calculated by Welch’s t-test (n = 21 HIV-negative, n = 18 aviremic, and n = 13 viremic HIV-positive subjects). Notch4 levels in HIV-positive plasma exosome fractions correlate negatively with increasing CD4/CD8 ratio and positively with K:T ratio and exosome-HLA-DRA levels in Pearson correlations (bottom panels, p < 0.05; n = 25–27).

HIV infection is associated with increased immune activation, reactive oxygen species (ROS), and oxidative stress^33,34^. Exosomes are proposed to influence immune activation through cell-to-cell communication, while oxidative stress enhances exosome release from stressed cells^30,35^. We therefore examined inter-relationships between plasma exosomes and metabolite changes related to immune activation and oxidative stress. Our previous studies characterizing the plasma metabolome of HIV-positive subjects on ART identified alterations in pathways involved in tryptophan catabolism, n-3 and n-6 polyunsaturated fatty acid (PUFA) metabolism, and mitochondrial function^36,37^. Based on these earlier findings, we focused on metabolites related to: 1) tryptophan catabolism (increased with immune activation)^38–42^; 2) methionine and cysteine metabolism (altered with oxidative stress)^43,44^; and 3) n-3 and n-6 PUFA metabolism (anti-inflammatory pathway). Metabolomic profiling of plasma from HIV-positive subjects and controls detected 655 endogenous metabolites, from which we selected 16 mapping to these pathways for further analysis (Supplementary Table S2). Unsupervised hierarchical clustering of these 16 metabolites distinguished HIV-positive versus HIV-negative subjects (Fig. 3a). Thirteen metabolites were significantly altered in both aviremic and viremic HIV-positive subjects vs. controls (fold-change (FC) >1.3, p < 0.05, false discovery rate (FDR) <0.10) (Supplementary Table S2). Metabolites altered in HIV-positive subjects included markers of oxidative stress (Fig. 3b, top; p < 0.01 for cysteine, cystine, oxidized cys-gly, heme, cysteinylglycine, cysteine s-sulfate, methioinine sulfone, N1-methyladenosine), n-3 and n-6 PUFA (Fig. 3b, bottom; p < 0.001 for eicosapentaenoate (20:5n3) (EPA), docosahexaenoate (22:6n3) (DHA), docosapentaenoate (22:5n3) and n-3 and n-6 docosapentaenoate (22:5n6) (DPA), and tryptophan catabolism (kynurenine:tryptophan (K:T) ratio, a metabolite marker of immune activation in HIV infection, see Fig. 4 below). Oxidative stress metabolites and K:T ratio showed positive correlations, while n-3 and n-6 PUFA showed negative correlations with exosome markers (CD9 and CD63) (Figs 3c and d and S4; p < 0.05). These results suggest that increased abundance of plasma exosomes in HIV patients is associated with oxidative stress, immune activation, and reduced n-3 and n-6 PUFA.

### Proteomic analysis and detection of Notch4 in plasma exosomes

To further characterize plasma exosomes from HIV-positive and healthy control subjects, isolated exosome fractions were subjected to untargeted LC/MS/MS proteomics by 2 different methods after depletion of abundant plasma proteins such as albumin and immunoglobulins and immunoaffinity purification (IAP) using antibodies against exosome markers CD9, CD63, and CD81 (Supplementary Figure S5). In the first method, tryptic digestion and LC/MS/MS analysis of IAP-purified exosomes from one HIV-positive and one HIV-negative subject identified peptides mapping to 80 unique proteins (Supplementary Table S3), of which 34 proteins were previously shown to be exosome-associated (exocarta.org) and 35 were classified as abundant plasma proteins. The second method analyzed IAP-purified exosomes from 2 HIV-negative or 3 HIV-positive pooled donors, respectively, following tryptic digestion and LC/MS/MS on a more sensitive instrument, and detected 321 proteins (Supplementary Table S4), of which 217 were previously shown to be exosome-associated (exocarta.org) and 165 were classified as abundant plasma proteins. GO mapping of exosome-associated proteins detected in these experiments identified proteins associated with myeloid cells (CD14, CSF1R), immune activation/inflammation (ADAM33, BPIFA1, BP1FB1, CAMP, CRP, CPN1, HLA-A, HLA-B, ITGB1, LILRB1, LILRB2), transmembrane signaling (EFNA4, ITGA6, LRP8, NOTCH4), microvesicles/exosomes (CD9, CD63, CD81, CDH1, ENO1, RAB1A, SDCBP), oxidative stress (CAT, ENO1, PRDX1, PRDX2, SEPP1, TXN), stress responses (HSPA5, RAC1, WARS), and platelets (CD36, FERMT3, ITGA2B, ITGB3) (Table 2). Notably, the immune activation markers CD14, CRP, HLA-A, and HLA-B were detected only in HIV-positive plasma exosomes.

**Table 2:** Biological classification based on gene ontology annotation of proteins identified by mass spectrometry analysis of immunoaffinity-purified plasma exosomes.

Biological classification	HIV-negative (n = 3)	HIV-positive (n = 4)
immune activation/inflammation	ADAM33, **AZGP1**, **BPIFA1, BPIFB1**, BTN2A2, CAMP, CD4, CSF1R, CST6, CXCL16, **DDR1**, DMBT1, **ENO1**, **FERMT3, LILRB1**, LILRB2, LYZ, **PLTP, TFRC**	**ADAM33, BPIFB1, BTN2A2, CAMP, CD14, COLEC10, CPN1**, **CRP**, **CSF1R, DDR1, ENO1**, **FERMT3, HLA-A, HLA-B**, **ITGB1**, ICAM2, **LILRB1, PLTP**, **TFRC**
transmembrane signaling	CD4, EFNA4, LRP8, **NOTCH4**, PTRF, **TFRC**	**EFNA4**, **ITGA6, LRP8, NOTCH4**, **TFRC**
extracellular vesicles/exosomes	**ANXA2, APMAP, AZGP1, BPIFB1, CAMP, CAT**, CD9, CD63, CD81, CDH1, **ENO1, FERMT3, FLNA, GAPDH, HSPA5**, LAMP1, LDHA, **MYH9, PFN1, PKM, PRDX1, PRDX2, PXDN, RAP1A, SDCBP, TXN, UBA52, YWHAB, ZG16B**	**ADAM10, APMAP, ARF1, BPIFB1, CAMP, CDH1**, CDC42, **CRTAC1, ENO1, FERMT3, GAPDH, GPLD1, LAMP1, MYH9, PCYOX1, PKM**, RAB1A, **RAC1, RAP1A, SDCBP**, SPTAN1**, STOM, TLN1, UBA52**
stress response	CAT, CDH1**, ENO1**, **GAPDH, HSPA5**, HSPA1L, **PRDX1**, **PRDX2, TXN**, TYMP, WARS	**ADAM10**, BMP1, **CDH1, ENO1**, **GAPDH**, MAP3K11, **RAC1, SEPP1**
oxidative stress	CAT, **ENO1, GAPDH, PRDX1**, **PRDX2, PXDN**, TPM1, TXN, WARS	**CRP, ENO1, GAPDH**, GPX3, **ITGB1**, **RAC1**, SLC25A33, **SEPP1**
fatty acid/lipid metabolism	**AZGP1**, **CD36**, **CETP**, DPEP3, FABP5, HGFAC, LRCOL1, **PKM, PLTP**	ACOX3**, ADIPOQ**, ANGPTL3, **CD36**, **CETP, DPEP3, GPLD1**, HADHA**, HGFAC, LRCOL1, PCSK9, PKM, PLTP**, SAR1A
platelets	**CD36, FERMT3, ITGA2B**, ITGB3, MMRN1	**CD36, FERMT3, F11R, ITGA2B, ITGB3**, PF4V1

PANTHER and Biobase TRANSFAC tools were used for gene ontology (GO) mapping of proteins identified by mass spectrometry analysis of IAP-purified plasma exosomes from 3 HIV-negative and 4 HIV-positive subjects in the merged datasets shown in Supplemental Tables 3 and 4. GO groups were assigned to the indicated biological functions. Individual proteins may be annotated to more than one category. Proteins identified by 2 or more unique peptides are shown in bold.

To confirm proteins of interest detected by proteomics using another method, we performed immunoblotting to probe 3 proteins that could potentially play a functional role in immune activation signaling (ADAM10, DDR1, Notch4) in exosome samples. Among these candidates, only Notch4 was detected by immunoblotting of these plasma exosome fractions (Fig. 4, top left). Therefore, further studies focused on Notch4. Notch4 levels in plasma exosomes from HIV-viremic subjects were higher than those in HIV-aviremic and HIV-negative subjects based on ELISA (Fig. 4, top middle panel) and immunoblotting (data not shown). Although Notch4 levels increased with increasing EV numbers, the correlation did not reach statistical significance (p = 0.07, Supplementary Figure S6). K:T ratio was increased in both aviremic and viremic HIV-positive subjects (Fig. 4, top right panel). Plasma exosome-associated Notch4 correlated positively with K:T ratio and exosome-associated-HLA-DRA measured by ELISA (Fig. 4, bottom middle and right panels) and negatively with CD4/CD8 ratio, an indicator of poor CD4 T cell recovery associated with increased immune activation (Fig. 4, bottom left). Thus, Notch4 is a novel plasma exosome-associated protein identified by untargeted proteomics that correlates with immune markers and exosomal HLA-DRA.

### Notch4 is expressed in human dendritic cells

**Figure 5:**
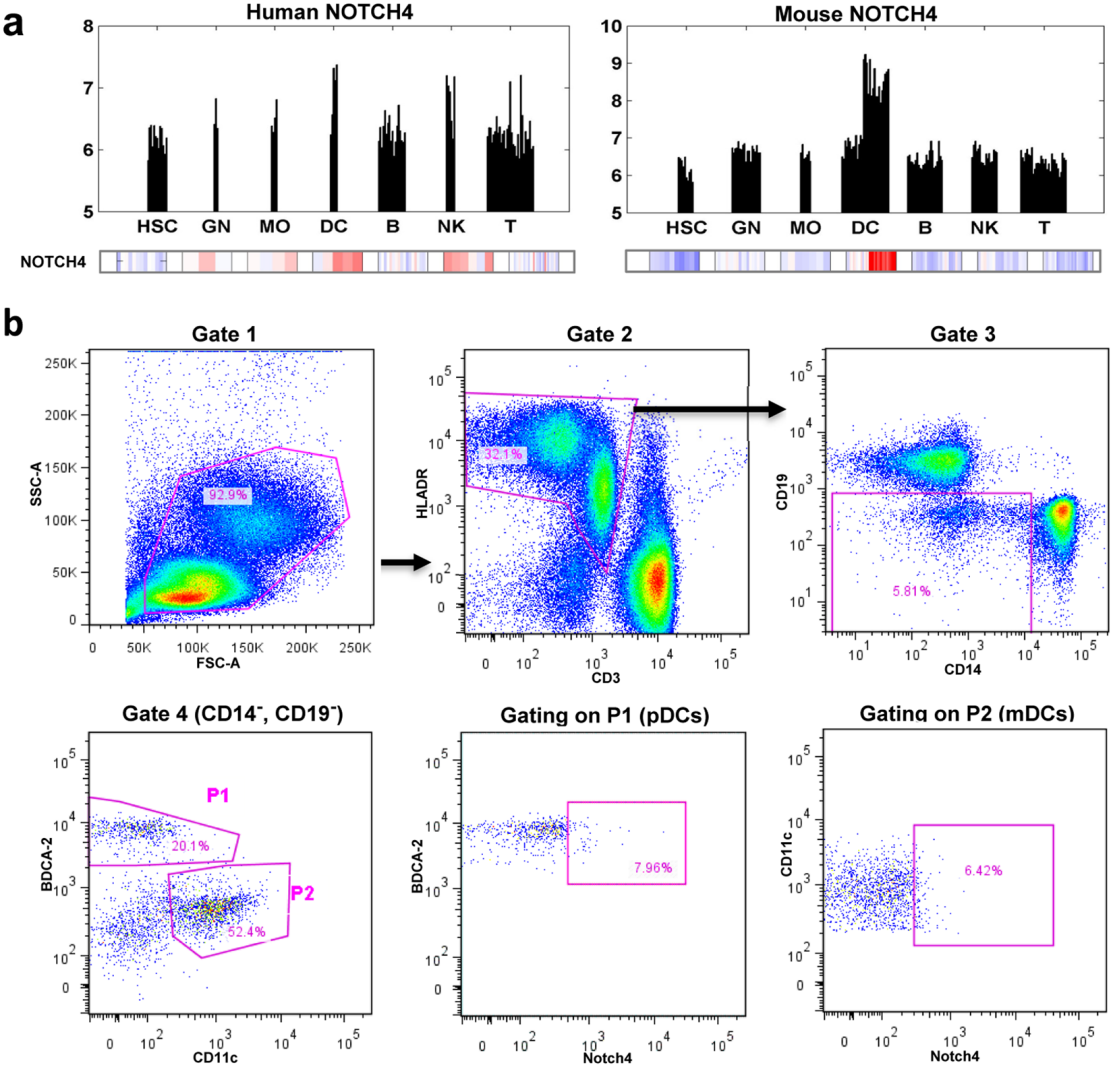
Notch4 is expressed in human dendritic cells. (**a**) Notch4 gene expression profiles from Affymetrix microarray profiling of human and mouse immune cell lineages from different individual human donors (left) or different pools of C57BL/6 (B6) mice (right) from the ImmGen Consortium Database (www.immgen.org). Bar plots (top panel) show absolute NOTCH4 mRNA expression in individual cell lineages in the indicated cell types. Heatmaps show mean-centered gene expression values (red bars denote enrichment above background). NOTCH4 mRNA is expressed in dendritic cells in both human and mouse, whereas expression in NK cells is detected only in humans. (**b**) Representative density plots from flow cytometric analysis show gating strategy and expression of Notch4 in plasmacytoid and myeloid dendritic cell subpopulations in PBMCs (one representative donor from a total of n = 3 healthy donors). HSC, Hematopoietic stem cells; GN, Neutrophils; MO, Monocytes; DC, Dendritic cells; B, B lymphocytes; NK, Natural killer cells; T, T lymphocytes.

**Figure 6:**
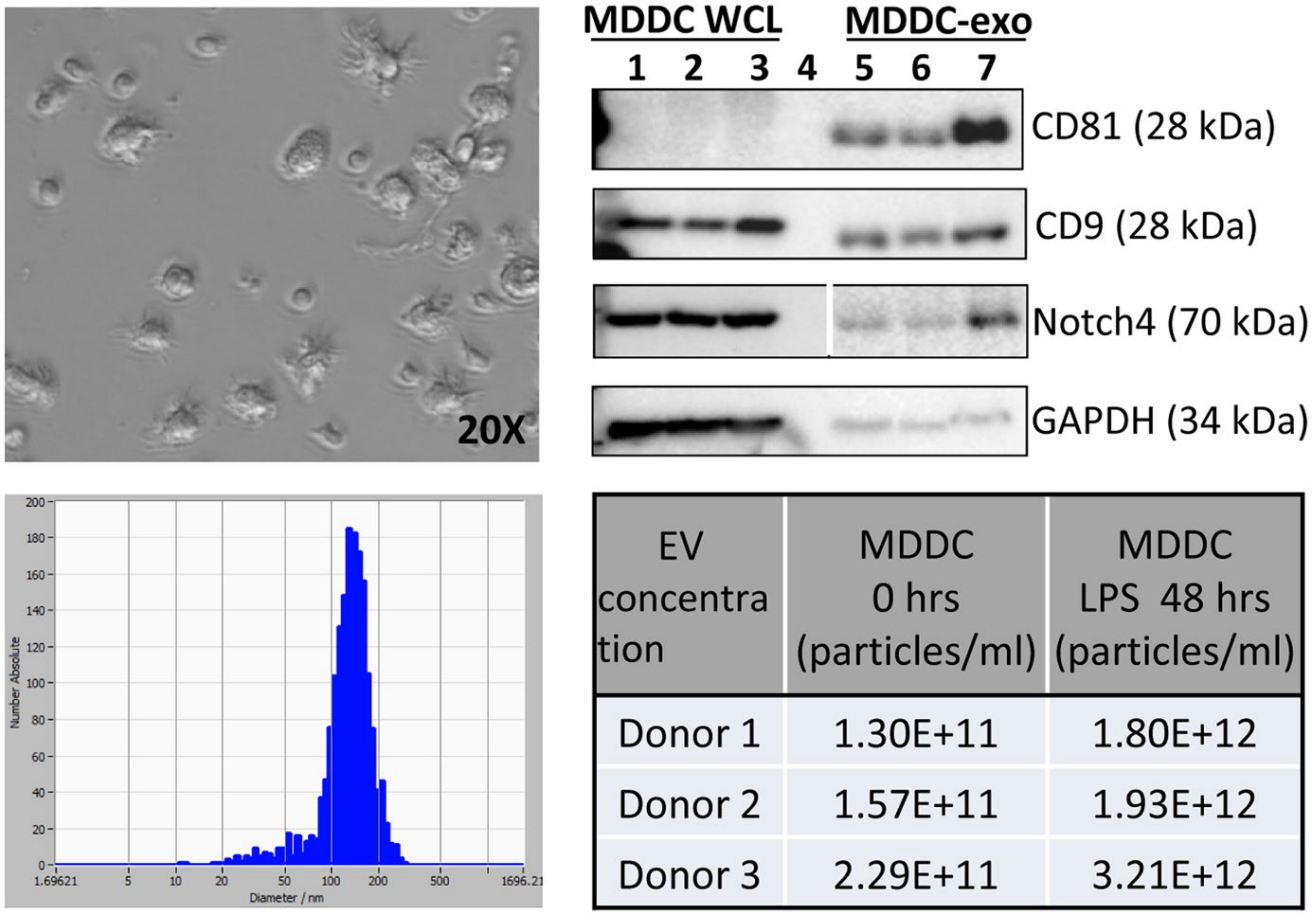
Exosomes released from cultured MDDCs carry Notch4 protein. MDDCs (from n = 3 healthy donors) cultured for 5 days with exosome-depleted FBS were treated with LPS (100 ng/ml) for additional 48 hrs to induce maturation (top left panel). Exosomes were isolated from culture supernatants of mature MDDC; EV concentration and size distribution was measured before and after (48 hrs) LPS treatment, by nanoparticle tracking analysis (bottom panel). Protein extracted from mature MDDC whole cell lysates and exosomes (30 μg/lane) was immunoblotted for exosome markers, Notch4, and GAPDH (top right). Lanes 1–3 = whole cell lysates, lane 4 = PBS blank, lane 5–7 = mature MDDC derived exosomes. Blots are cropped and full-length blots are included in Supplementary Figure S7.

Next, we investigated potential sources of plasma exosomes carrying Notch4 protein. Notch4 mRNA is highly expressed in human dendritic cells and natural killer (NK) cells based on gene expression profiles of different immune cell populations from the Immgen Consortium Database (www.immgen.org), suggesting these immune cells are a potential source of exosomal Notch4 (Fig. 5, top panel). To further investigate this possibility, we used flow cytometry with antibodies against Notch4 and immune cell markers to assess staining on freshly isolated PBMCs from 3 randomly selected healthy donors. Notch4 staining was detected in a minority of plasmacytoid and myeloid dendritic cells (Fig. 5, middle and bottom panels), and although it can also be expressed on monocytes, NK cells, or lymphocytes it is generally at much lower levels (data not shown). Thus, circulating dendritic cells are one potential source of exosomes carrying Notch4. To confirm this, we generated monocyte-derived dendritic cells (MDDC) *in vitro* from 3 randomly selected healthy donors, induced maturation with LPS treatment (Fig. 6, top left)^45^, and isolated exosomes from MDDC conditioned medium before and after LPS treatment to activate MDDCs and mimic immune activation conditions^2^. NTA analysis showed an increase in EV concentrations after LPS treatment in all 3 donor samples (Fig. 6, bottom panels). Immunoblotting was performed on mature MDDC-whole cell lysates (WCL) and exosome fractions for exosome markers, Notch4, and GAPDH (Fig. 6, top right). Exosome markers (CD9 and CD81) and Notch4 levels in exosome fractions, but not WCL fractions, increased with increasing EV numbers based on western blot band intensities. These results suggest that dendritic cells are a potential source of Notch4 in circulating plasma exosomes.

### Immunomodulatory effects of plasma exosomes on THP-1 monocytes

**Figure 7:**
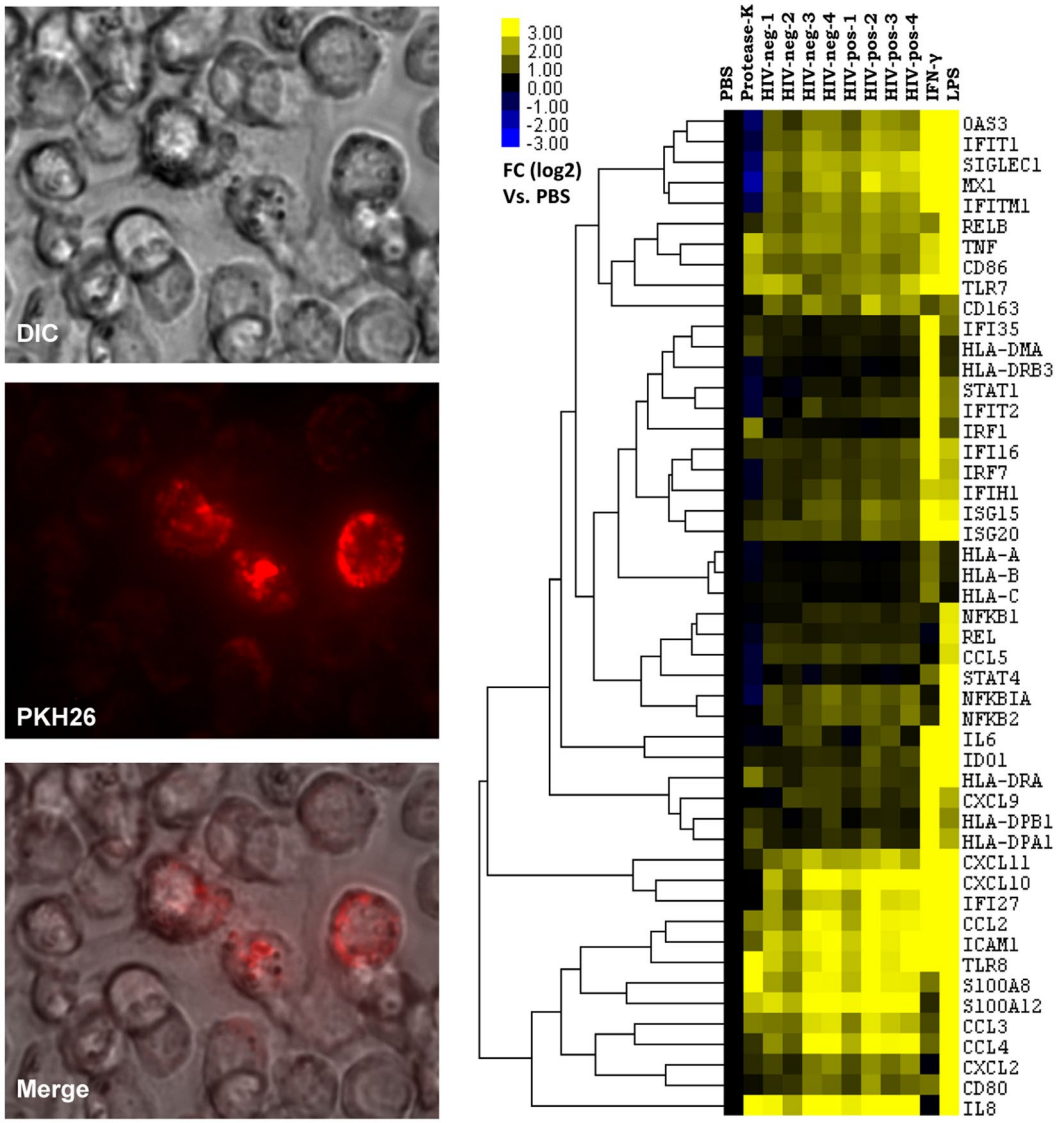
Plasma exosomes from HIV-positive subjects induce gene expression changes indicative of immunomodulatory effects and immune activation in THP-1 monocytic cells. THP-1 cells were treated with 20 μg PKH26-labeled plasma exosomes for 72 hrs (left panel). THP-1 cells were treated with immunoaffinity purified-plasma exosomes (20 μg) from 4 HIV-positive and 4 HIV-negative subjects for 72 hrs (lanes 3 to 10) or with IFN-γ (100 IU/ml) or LPS (0.5 μg/ml) for 24 hrs (lanes 11 and 12). Gene expression was measured using the Nanostring nCounter PanCancer Immune Profiling Panel. Heatmap shows unsupervised hierarchical clustering of expression changes in 49 genes associated with interferon response, cytokines/chemokines, immune activation, innate immune response, and inflammation following treatment with exosomes relative to controls (lanes 1 and 2). Treatment of THP-1 cells with HIV-positive and HIV-negative plasma exosomes induced gene expression changes partially overlapping those induced by IFN-γ and LPS. Representative of 2 independent experiments.

Exosome uptake has diverse effects on recipient cells, depending on their cellular source, specific cargo, disease or stress conditions, and recipient cell types. To evaluate effects of exosome uptake *in vitro* on gene expression profiles, THP-1 monocytic cells, which are commonly used as a cell culture model in exosome studies^14,25,46^, were treated with exosomes isolated from plasma of HIV-positive and control subjects. Initial experiments were performed to optimize the time required for exosome uptake. THP-1 cells were incubated with PKH26 dye-labeled plasma exosomes for 48 or 72 hrs, which indicated optimal exosome uptake at 72 hrs (Fig. 7, left panel). THP-1 cells were incubated for 72 hrs with IAP-purified plasma exosomes from HIV-aviremic and age-matched control subjects (n = 4 per group); THP-1 cells treated with PBS, or Proteinase K-shaved exosomes, served as negative controls. IFN-γ (100 IU/ml) or LPS (0.5 μg/ml) treatment for 24 hrs served as positive controls. Gene expression profiling of THP-1 total RNA using Nanostring technology was used to probe 800 genes, from which we selected 80 genes for further analysis based on known relationships to monocyte/macrophage activation, inflammation, innate immune responses, and interferon responses (Supplementary Table S5). Forty-nine genes were selected for heatmap visualization after excluding 31 genes with minimal expression across all samples (Fig. 7, right panel). Treatment of THP-1 cells with IAP-purified plasma exosomes from HIV-positive and HIV-negative subjects induced gene expression changes that partially overlapped those induced by IFN-γ and LPS. The genes showing maximal transcriptional activation were related to interferon responses (IFIT1, IFITM1, IFI27, ISG15, ISG20, MX1, OAS3), cytokines/chemokines (CCL2, CXCL2, CXCL10, CXCL11, TNF, IL8), immune activation (CD80, CD86), innate immune responses (S100A12, IFIH1, TLR7, TLR8, NFKB1A, NFKB2, ICAM1), and inflammation (S100A8). Two of four control plasma exosome samples (HIV-negative subjects 3 and 4) induced gene expression patterns similar to those elicited by HIV-positive plasma exosomes. These data suggest that exosomes from plasma of HIV-positive subjects treated with ART carry cargo related to inflammation and immune activation, and have immunomodulatory effects on myeloid cells.

## Discussion

In this study, we show that HIV-positive subjects on ART have higher abundance of circulating plasma exosomes as compared to uninfected controls matched for demographics and smoking. The exosome markers CD9, CD63, and HSP70 were elevated in plasma from HIV-positive subjects on ART; CD9 and CD63 were elevated even in aviremic HIV-positive subjects. These findings contrast to a previous study by Hubert *et al*.^7^, which showed that circulating plasma exosomes are more abundant (as measured by an indirect assay using acetylcholinesterase activity) in ART-naive, but not ART-suppressed subjects, relative to healthy controls. Contrary to expectations, EV numbers or exosome markers such as CD9 and CD63 did not correlate with plasma VL, CD4 counts, and CD4:CD8 ratio in HIV-positive subjects on ART. This finding is consistent with Hubert *et al*.^7^, which reported that exosome abundance correlated with CD4 counts in ART-naive HIV-positive subjects with high VL (mean 10,000 HIV RNA copies/ml), but not in ART-suppressed subjects. Our findings suggest that circulating exosomes are increased in patients with treated HIV infection, but the levels do not show a clear relationship to virological and immunological parameters in treated patients with suppressed VL.

Our previous studies identified changes in plasma metabolites related to chronic immune activation and inflammation in HIV patients on ART^36,37^. Here, we evaluated plasma metabolites using a more sensitive LC/MS/MS platform and detected significant increases in metabolites associated with oxidative stress (cysteine, cystine, oxidized cys-gly, heme, cysteinylglycine, cysteine s-sulfate, methioinine sulfone, N1-methyladenosine), immune activation (K:T ratio), and decreased n-3 and n-6 PUFA (DHA, EPA, n-3 and n-6 DPA). These anti-inflammatory PUFA are reduced in HIV-infected individuals compared to healthy controls^36^, which may influence progression to AIDS and comorbidities^47^. Moreover, decreased n-3 and n-6 PUFA can render cells more susceptible to oxidative stress and ER-stress, leading to cell damage and apoptosis^48^. Increased ROS and oxidative stress are detected in HIV-infected individuals on ART compared to those not receiving treatment or uninfected healthy controls^34,49^. Oxidative stress increases exosome secretion *in vitro*, which can communicate protective messages to other cells mediated in part by exosomal shuttling of RNA cargo^30,50^. K:T ratio, a metabolite marker of immune activation, is increased in the setting of HIV infection^40^. K:T ratio was increased in HIV patients in our study cohort, and increasing exosome abundance correlated positively with this immune activation marker. Our finding that exosome markers correlate positively with increasing oxidative stress metabolites and immune activation markers, and carry protein cargo related to oxidative stress and immune activation (e.g., catalase, peroxiredoxins, enolase, selenoprotein P, HLA-A, HLA-B, ADAM10, CRP) are consistent with functional roles for exosomes in redox homeostasis and immunological functions.

Several studies reported effects of HIV infection on exosome secretion, abundance, and cargo *in vitro*^22,25,51^. Although some studies suggested that circulating exosome cargo may be related to chronic inflammation in HIV infection^7,8^, few studies have investigated circulating exosomes as biomarkers. By untargeted proteomics of exosomes immunoaffinity-purified from plasma of HIV-positive subjects on ART and healthy controls, we identified exosome protein cargo related to myeloid cells, immune activation, inflammation, and oxidative stress. Of 321 exosome-associated proteins detected by proteomics, at least 40 were related to immune activation/inflammation, of which 9 were detected only in HIV-positive exosomes including CD14, CRP, HLA-A, HLA-B, and ITGB1. CRP is an inflammation marker secreted by the liver in response to IL-6 stimulation^52^. To our knowledge, this is the first study detecting CRP in exosomes, although monomeric CRP has been detected in circulating microparticles in the setting of myocardial infarction^53^. CRP levels are higher in individuals with HIV infection, often rising over time^54^ in association with HIV disease progression^55^. We identified CD14 in plasma exosomes, suggesting that myeloid cells are a likely source of circulating exosomes; however, platelets are another potential source based on our detection of platelet-associated markers in exosome fractions. Another category of proteins detected in exosomes were proteins related to oxidative stress including CAT, ENO1, PRDX1, PRDX2, PXDN, SEPP1, and TXN. These proteins are involved in reducing oxidative stress, and were detected more frequently in HIV-negative exosomes.

We show here, for the first time, the presence of Notch4 in plasma exosomes. Notch4 is a receptor for Jagged-1, Jagged-2, and Delta-1, and regulates cell-fate determination. The intracellular domain is activated by proteolytic cleavage and translocates to the nucleus, where it forms a transcriptional activator complex. While Notch1, 2 and 3 have been previously detected in exosomes^56–59^, our study is the first to provide evidence for Notch4 in plasma exosomes by LC-MS/MS and immunoblotting. Furthermore, we provide evidence that myeloid and plasmacytoid dendritic cells are one potential source of Notch4-containing exosomes in plasma based on gene expression and flow cytometry, which detected Notch4 expression in myeloid and plasmacytoid dendritic cells, and correlation between exosomal Notch4 and HLA-DRA levels. Notch signaling is critical for differentiation, development, and functions of dendritic cells, including plasmacytoid dendritic cells responding to viral stimulation by IFN-α secretion^60–62^. Dendritic cell secretion of cytokines/chemokines and downstream polarization of Th-type responses are also regulated by Notch signaling^63^. Notch4 is expressed by endothelial cells^64^, which represent another potential source of Notch4-containing exosomes. However, we assayed exosome fractions for endothelial marker CD105 by ELISA and found no correlation with exosomal Notch4 levels (data not shown); examination of other potential sources requires further study. Exosomal Notch4 correlated with increased K:T ratio, decreased CD4/CD8 ratio, and exosomal HLA-DRA, which are indicators of immune activation in HIV infection^41,65,66^. Furthermore, Notch4 was detected in exosomes released from mature MDDCs following treatment with LPS *in vitro*. These findings suggest that exosomal Notch4 is a potential biomarker of immune activation in HIV infection.

Previous studies show immunomodulatory effects in monocytic cells induced by exosomes^14^. We therefore hypothesized that plasma exosomes of HIV-positive individuals on ART may have immunomodulatory effects on recipient cells. We tested this hypothesis using a cellular model in which THP-1 monocytes were incubated with IAP-purified plasma exosomes from HIV and control subjects; changes in gene expression were examined using the NanoString platform, which utilizes digital color-coded barcodes to detect and count hundreds of unique transcripts in a single reaction, without PCR amplification^67^. Genes associated with interferon responses, innate immune responses, and inflammation, were upregulated in THP-1 cells following incubation with exosomes; many genes induced by exosome treatment were also induced by IFN-γ and/or LPS. These findings suggest that circulating exosomes can have pro-inflammatory and other immunomodulatory effects on recipient cells. However, these effects were not specific to plasma exosomes from HIV-positive subjects: in 2 of 4 healthy control donors, plasma exosomes induced gene expression changes similar to those induced by HIV-positive exosomes. Triggering of cell signaling and gene expression changes are not unique to exosomes from diseased/stressed conditions^14,68^. We cannot exclude the possibility that modulation of some analyzed genes was the result of secondary events subsequent to exosome stimulation of THP-1 cells. Nonetheless, we found that HIV-positive exosomes had stronger effects on recipient cells compared to HIV-negative exosomes.

We acknowledge limitations of the study, particularly those related to the purity of exosome preparations^69^. Although plasma is a good source of exosomes, it is challenging to separate plasma exosomes from abundant plasma proteins, larger microvesicles, subcellular fractions, and cholesterol particles. Our study was also limited by small volumes of plasma available for exosome isolation. Exosome isolation by differential ultracentrifugation yielded exosome fractions of higher purity than the Exoquick method, but required larger starting volumes (>1 ml) and pooling of multiple samples. Importantly, both methods gave similar results with regard to our main finding that exosome markers are increased in HIV-positive compared to HIV-negative plasma. The optimal method to isolate exosomes from small volumes of plasma is to precipitate exosomes using an exosome-precipitating reagent to minimize exosome loss, and then purify the exosome fraction by another method such as immunoaffinity purification. For proteomics, we purified plasma exosome fractions by depleting 12 abundant plasma proteins, followed by IAP using exosome marker antibodies. This method resulted in higher purity of exosome fractions, but lower exosome yield. Another limitation of our study was lack of availability of primary DCs from HIV-infected patients, which would be more relevant to study sources of Notch4-bearing exosomes during HIV infection. Likewise, future studies using primary monocytes would be helpful to validate our findings from THP-1 cells. Additional limitations of our study include the relatively small sample size and high rates of comorbidities such as smoking, HCV-coinfection, and cocaine or alcohol abuse, which could influence some findings. High rates of smoking and cocaine use in our study cohort may have influenced markers of inflammation and oxidative stress^70–74^, but we did not have sufficient statistical power to evaluate effects of these covariates. Further studies are needed to address the impact of these and other lifestyle factors on exosomes.

In conclusion, our study shows that: (1) HIV-positive individuals have elevated abundance of plasma exosomes and exosome markers (CD9, CD63 and HSP70), and these changes correlate with metabolites indicative of oxidative stress and immune activation; (2) Notch4 protein is detected in plasma exosomes, and correlates with immune activation markers in HIV-positive subjects; (3) plasmacytoid and myeloid dendritic cells express Notch4 mRNA and protein, and are one potential source of exosomes in plasma of HIV-positive subjects; and (4) circulating exosomes in ART-treated HIV-positive subjects carry protein cargo related to immune activation and oxidative stress, have immunomodulatory effects on myeloid cells, and may have pro-inflammatory and redox effects during HIV pathogenesis. Understanding the relationship of exosome cargo to HIV pathogenesis, immune activation, and oxidative stress may contribute to novel biomarker discovery, elucidate mechanisms contributing to disease progression and comorbidities, and accelerate the identification of new biomarkers and therapeutics.

## Methods

### Study subjects

The study was performed in accordance with guidelines in the Declaration of Helsinki. Plasma samples from HIV-positive subjects (n = 43, age 35–65 years) were from the National NeuroAIDS Tissue Consortium (NNTC) [Manhattan HIV Brain Bank (n = 8), National Neurological AIDS Bank (n = 4), California NeuroAIDS Tissue Network (n = 12), Texas NeuroAIDS Research Center (n = 3)] and AIDS Linked to the Intravenous Experience (ALIVE) cohort (n = 16). All subjects were enrolled with written informed consent and IRB approval at each study site (IRB committees at Mount Sinai School of Medicine and Mount Sinai Hospital, UCLA, University of California San Diego, and Johns Hopkins Bloomberg School of Public Health, respectively). Inclusion criteria for HIV-positive subjects were: combination ART use with HIV plasma VL undetectable or below 2500 HIV RNA copies/ml. For NTA measurements only, we included 4 HIV-positive subjects not reporting ART use at time of plasma collection (plasma VL range, 507 to 2,405 HIV RNA copies/ml). Exclusion criteria were diagnoses or lab values indicative of renal or liver failure. Healthy control plasma samples (n = 34), were from HIV- negative donors (from Bioreclamation IVT, n = 29; ALIVE cohort, n = 5) with informed consent and IRB approval from Dana-Farber Cancer Institute. The healthy control group was frequency-matched to the HIV-positive group by age, race, gender, and smoking (Table 1).

### Exosome isolation and quality assessment

#### ExoQuick method

Plasma samples (0.4 ml) were centrifuged for 15 minutes at 3000 × g. Exosomes were precipitated using ExoQuick exosome precipitating reagent (System Bioscience) per manufacturer’s instructions. Exoquick reagent contains volume-excluding polymer (polyethylene glycol) that precipitates EVs including exosomes, thereby forming EV aggregates which can be easily isolated by low-speed centrifugation^75^. First, plasma was incubated with thromboplastin D for 5 min at room temperature to de-fibrinate plasma. After centrifugation at 10,600 × g for 5 min, supernatant was mixed with ExoQuick reagent and incubated at 4 °C for 1 hr. The mixture was centrifuged at 1500 × g for 30 min and exosome pellet resuspended in PBS. The resulting exosome fraction was passed through 0.22 μm filter to remove EVs larger than exosomes.

#### Differential Ultracentrifugation method

Exosome fractions were isolated by differential ultracentrifugation as described^76^. Plasma samples were pooled from HIV-positive and HIV-negative subjects (n = 15 each, see Supplementary Figure S1). For each sample, 1.3 ml plasma was centrifuged at 500 × g and the supernatant was collected and diluted 1:2 with PBS. Diluted plasma was centrifuged at 12,000 × g for 30 minutes and the supernatant was passed through 0.22 μm filter to remove EVs larger than exosomes. The filtrate was further diluted with PBS such that plasma was finally diluted 1:5 in PBS. Diluted plasma was ultracentrifuged at 120,000 × g for 70 minutes at 4 °C. Supernatant was decanted and exosome pellet resuspended in 100 μL PBS.

#### Exosome morphology and concentration assessment

Exosome morphology was characterized^77^ by imaging exosome fractions on a Tecnai G^2^ Spirit BioTWIN Transmission Electron Microscope (TEM) equipped with an AMT 2k CCD camera at the Harvard University TEM core. Size distribution and concentration of EVs was measured by nanoparticle tracking analysis (NTA) on a ZetaView instrument (Particle Metrix). Zetasizer Nano (Malvern Instruments) was also used to assess EV size distribution (see Supplementary Figure S1)

### Western blot analysis and ELISA

Exosome fractions were lysed in lysis buffer (Triton X-100 1%, NaCl 150 mM, sodium deoxycholate 0.5%, Tris-HCL 50 mM, SDS 0.1%, pH 7.4) and protein content measured by BioRad DC protein assay. Twenty-five micrograms of protein were separated in each lane of Tris SDS polyacrylamide gels (4–12% gradient), and transferred onto PVDF membranes. Blots were blocked with 5% milk and probed overnight at 4 °C with primary antibodies against exosome markers CD9, CD63, CD81, and HSP70 (System Bioscience, # EXOAB-KIT-1), Notch4 (Cell Signaling Technology, # 2423), and GAPDH (Cell Signaling Technology, # 2118), followed by appropriate secondary antibodies for one hour; signal was developed by enhanced chemiluminescence (ECL). Images were captured using a BioRad ChemiDoc™ Imaging System. Bands in each lane were normalized to corresponding EV numbers: band intensity values were divided by log10 transformed values of the corresponding EV number (see Supplementary Table S1). ELISA was performed on exosome fractions using commercially available kits for Notch4 (LifeScience Biosystem, LS-F12176) and HLA-DRA (MyBiosource, MBS706908) per manufacturer’s instructions. For ELISAs, exosome fractions were treated with RapiGest surfactant to release exosome contents prior to loading into wells.

### Metabolomic profiling

Untargeted metabolomic profiling was performed by Metabolon (Durham, NC) combining three independent platforms: ultra-high performance liquid chromatography and tandem mass spectrometry (UHLC/MS2/MS) optimized for detection of acidic metabolites, UHLC/MS2/MS optimized for detection of basic metabolites, and gas chromatography (GC)/MS. Plasma samples (100 ul) were extracted using the MicroLab STAR system as described^78^. Briefly, protein was precipitated from plasma with methanol containing four standards to monitor extraction efficiency. The resulting supernatant was split into equal aliquots for analysis on the three platforms. Aliquots, dried under nitrogen, were subsequently reconstituted in 50 μL 0.1% formic acid in water (acidic conditions) or in 50 μL 6.5 mM ammonium bicarbonate in water, pH 8 (basic conditions) for the two UHLC/MS/MS analyses or derivatized to a final volume of 50 μL for GC/MS analysis using equal parts bistrimethyl-silyl-trifluoroacetamide and solvent mixture acetonitrile:dichloromethane:cyclohexane (5:4:1) with 5% triethylamine at 60 °C for one hour. Three types of controls were utilized: samples derived from pooled experimental samples served as technical replicates, extracted water samples served as blanks, and a cocktail of standards spiked into every analyzed sample allowed instrument performance monitoring. The UHLC/MS2/MS platform was based on a Waters ACQUITY UHPLC and Thermo-Finnigan LTQ mass spectrometer, which consisted of an electrospray ionization source and linear ion-trap mass analyzer. Derivatized samples for GC/MS were separated on 5% phenyldimethyl silicone columns, with helium as the carrier gas and a temperature ramp from 60 °C to 340 °C over a 16-minute period. Analysis was performed on a Thermo-Finnigan Trace DSQ fast-scanning single-quadrupole mass spectrometer operated at unit mass resolving power with electron impact ionization and a 50–750 atomic mass unit scan range. Compounds were identified by automated comparison of the ion features in the experimental samples to a reference library of over 4, 000 chemical standard entries that included retention time, molecular weight (m/z), preferred adducts, and in-source fragments as well as associated MS spectra and curated by visual inspection for quality control using software developed at Metabolon^79^.

### Proteomic analysis

To obtain purified exosome fractions for proteomic analysis, 12 abundant plasma proteins were immunodepleted from plasma using Proteome Purify™ 12 kit (R&D systems) and albumin was depleted with 2 rounds of albumin depletion using AlbuSorb™ - Albumin Depletion Kit (Biotech Support Group). Plasma was pre-cleared using Protein A/G PLUS-Agarose beads (Santa Cruz Biotechnology) and exosome fraction precipitated using ExoQuick. Exosome fractions were further purified by immunoprecipitation from plasma exosome fractions using antibodies against CD9, CD63, and CD81 (#EXOFLOW32A-CD81, −CD63, −CD9, System Biosciences and # EX-COM-SP, JSRmicro). Isolated exosomes were analyzed by mass spectrometry using two different platforms: (1) ABSciex 4800Plus MALDI-TOF/TOF mass spectrometer and (2) Thermo Scientific LTQ-Orbitrap ion-trap mass spectrometer. In experiments using the first platform, samples were processed as follows: following immunoprecipitation, exosome-conjugated beads were washed thrice and exosomes were eluted as described^80^ using low pH buffer (50 mM glycine, pH 3.0) followed by neutralization of pH with 1 M Tris-HCl (pH 8.0). To ensure that exosomes were disrupted, detergent extraction was employed using deoxycholate. Protein was precipitated using cold methanol, then reduced, alkylated, and digested overnight using trypsin. Samples were run on nanoflow LC in reverse phase on a 15 cm C18 PepMap column on an LC Packings/Dionex nanoflow LC, and mass spectrometry was done on an ABSciex 4800Plus MALDI-TOF/TOF mass spectrometer. For peptide mapping and protein identification, database searches were performed using ProteinPilot 4.5b (ABSciex, Framingham, MA). Protein identifications with at least 95% confidence as determined by ProteinPilot were considered significant. In experiments using the second mass spectrometry platform, samples were processed as follows: following immunoprecipitation, exosome-conjugated beads were washed thrice and exosomes were solubilized with 0.5% RapiGest and boiling at 100 °C for 5 min. Residual immunoglobulins were depleted with Protein A/G PLUS-Agarose beads. Proteins were then digested overnight using trypsin (Sequence grade; Promega). Samples were then dried in a speed-vac and rehydrated in 50 µl of 0.5% TFA. The sample was then run through a stage tip (C18 tip), washed, and eluted. The solution was dried and then rehydrated in 2.5% acetonitrile and 0.1% formic acid solution. Reverse-phase fractionation was done on a 25 cm C18 column and samples were run on LTQ-Orbitrap ion-trap mass spectrometer (ThermoFisher). Database search was performed using the software program, Sequest (ThermoFisher). Common contaminants (keratin, bovine proteins, mouse/rabbit IgGs) were omitted from downstream analysis. Remaining proteins identified were compared against existing exosome database (www.exocarta.org) and the top 200 most abundant plasma proteins published previously^81^. Functional annotation was performed by GO mapping using PANTHER (pantherdb.org) and Biobase (genexplain.com/transfac).

### MDDC culture and exosome isolation

PBMCs were isolated from blood samples of 3 healthy donors using Histopaque-1077 (Sigma). Fresh PBMCs were seeded (6 × 10^6^ cells/ml) in 30 ml RPMI 1640 media supplemented with 10% FBS (depleted of exosomes by ultracentrifugation at 100,000 × g) and 1% Penicillin-Streptomycin. Following monocyte enrichment by plastic adherence (8–10% cells attached), differentiation to MDDC was induced by GM-CSF (800 U/ml) and IL-4 (500 U/ml) treatment^82^. After 5 days in culture, MDDCs were treated with LPS (100 ng/ml) for an additional 48 hrs to induce maturation^45^. Conditioned media was collected from mature MDDC, and centrifuged at 300 × g for 5 min and 3000 x g for 15 min. The supernatants were passed through 0.22 μm filter, and exosomes were then isolated using Exoquick TC (System Bioscience) per manufacturer’s instructions. The exosome pellet was re-suspended in 40 µl PBS.

### Polychromatic Flow Cytometry analysis of PBMCs

PBMCs were isolated from healthy donor blood samples using Histopaque-1077 (Sigma). Fresh PBMCs (1 × 10^6^ cells) were stained with LIVE/DEAD Aqua dye (Life Technologies) for 30 min at room temperature. Cells were then stained with anti-CD3, anti-CD4, anti-CD8, anti-CD56, anti-CD16, anti-HLA-DR, anti-Notch-4, anti-BDCA-1, anti-BDCA-2, anti-CD11c, anti-CD19, and anti-CD14 (Supplementary Table S6) and fixed with 1% paraformaldehyde. Data were acquired in an LSR II flow cytometer (BD Biosciences) and analyzed using FlowJo v10 software (Tree Star Inc., Ashland, OR).

### Functional analysis of plasma exosomes in THP-1 recipient cells

THP-1 suspension cells were cultured in 6-well plates at 2.5 × 10^5^ cells/ml in RPMI 1640 media supplemented with 1 mM sodium pyruvate, 10% FBS (depleted of exosomes by ultracentrifugation at 100,000 × g) and 1% penicillin-streptomycin. Cells were treated for 72 hrs with IAP-purified plasma exosomes (20 μg) from HIV-positive (n = 4) and healthy controls (n = 4). THP-1 cells treated with PBS and “shaved exosomes” pre-treated with Proteinase-K (to shave off surface proteins) were used as negative controls^83^. Proteinase K was inactivated by incubation at 70 °C for 15 min. Prior to exposing THP-1 cells to “shaved exosomes”, cells were treated with Proteinase K for 30 minutes and washed twice with PBS to remove excess Proteinase K. THP-1 cells were treated with the exosomes for 72 hrs. THP-1 cells treated with IFN-gamma (100 IU/ml) or LPS (0.5 μg/ml) for 24 hrs served as positive controls. Treated cells were collected and rinsed in PBS. Total RNA was isolated using the MirVana kit (Thermo Fisher Scientific). RNA content and quality was evaluated by BioAnalyzer (Agilent). mRNA hybridization, detection, and scanning were performed on 100 ng of total RNA using NanoString Counter technology^84^ with probes for 770 genes in the PanCancer Immune Profiling Panel and 30 PLUS custom probes (NanoString Technologies, Seattle, WA) at the DFCI Molecular Biology core facility.

### Data processing, bioinformatics, and statistical analysis

For metabolite profiling, metabolite data was normalized by median centering. Missing values were imputed with the lower limit of detection for a given metabolite. Batch normalization was performed using the median ratio for each metabolite in duplicate “anchor” samples across runs. Significantly altered metabolites were defined by FC > 1.3, p-value < 0.05, and FDR < 0.10. Statistical analyses using Welch’s t-test (p < 0.05) were performed on log-transformed data. Pearson correlations were used to evaluate relationships between plasma metabolites and exosome marker levels (p < 0.05). Metabolite clusters were identified by unsupervised hierarchical clustering using the heatmap.2 function of R. For Nanostring gene expression profiling, raw counts were normalized after quality control-check, and fold-change calculations and heatmaps were constructed using nSolver 3.0 software. Significantly altered genes were defined by FC > 1.3, p-value < 0.05 and FDR < 0.10. False-discovery rates for metabolite and gene expression profiling were estimated using fdrtool in R. Exosome marker levels were compared between groups using the Mann Whitney U-test in PRISM (p < 0.05).

### Data availability

All data generated or analyzed during this study are included in this published article (and its Supplementary Information files) or available from the corresponding author on reasonable request.

## Electronic supplementary material

Supplementary Information

Supplementary Table S1

Supplementary Table S2

Supplementary Table S3

Supplementary Table S4

Supplementary Table S5

## References

[1] Deeks SG, Tracy R, Douek DC (2013). Systemic effects of inflammation on health during chronic HIV infection. Immunity.

[2] Brenchley JM (2006). Microbial translocation is a cause of systemic immune activation in chronic HIV infection. Nature medicine.

[3] d’Ettorre G, Paiardini M, Ceccarelli G, Silvestri G, Vullo V (2011). HIV-associated immune activation: from bench to bedside. AIDS Res Hum Retroviruses.

[4] Boukouris S, Mathivanan S (2015). Exosomes in bodily fluids are a highly stable resource of disease biomarkers. Proteomics Clin Appl.

[5] De Toro J, Herschlik L, Waldner C, Mongini C (2015). Emerging roles of exosomes in normal and pathological conditions: new insights for diagnosis and therapeutic applications. Front Immunol.

[6] Greening DW, Gopal SK, Xu R, Simpson RJ, Chen W (2015). Exosomes and their roles in immune regulation and cancer. Semin Cell Dev Biol.

[7] Hubert, A. *et al*. Elevated abundance, size and microRNA content of plasma extracellular vesicles in viremic HIV-1 + patients: correlations with known markers of disease progression. *J Acquir Immune Defic Syndr*, 10.1097/QAI.0000000000000756 (2015). 10.1097/QAI.0000000000000756 PMC4627170 26181817

[8] Konadu KA (2015). Association of Cytokines With Exosomes in the Plasma of HIV-1-Seropositive Individuals. J Infect Dis.

[9] Madison MN, Okeoma CM (2015). Exosomes: Implications in HIV-1 Pathogenesis. Viruses.

[10] Teow SY, Nordin AC, Ali SA, Khoo AS (2016). Exosomes in Human Immunodeficiency Virus Type I Pathogenesis: Threat or Opportunity?. Adv Virol.

[11] Yanez-Mo M (2015). Biological properties of extracellular vesicles and their physiological functions. Journal of extracellular vesicles.

[12] Robbins PD, Morelli AE (2014). Regulation of immune responses by extracellular vesicles. Nat Rev Immunol.

[13] Thery C, Ostrowski M, Segura E (2009). Membrane vesicles as conveyors of immune responses. Nat Rev Immunol.

[14] Bretz NP (2013). Body fluid exosomes promote secretion of inflammatory cytokines in monocytic cells via Toll-like receptor signaling. The Journal of biological chemistry.

[15] Longatti A (2015). The Dual Role of Exosomes in Hepatitis A and C Virus Transmission and Viral Immune Activation. Viruses.

[16] Lenassi M (2010). HIV Nef is secreted in exosomes and triggers apoptosis in bystander CD4 + T cells. Traffic.

[17] Nguyen DG, Booth A, Gould SJ, Hildreth JE (2003). Evidence that HIV budding in primary macrophages occurs through the exosome release pathway. The Journal of biological chemistry.

[18] Izquierdo-Useros N (2010). HIV and mature dendritic cells: Trojan exosomes riding the Trojan horse?. PLoS Pathog.

[19] Kadiu I, Narayanasamy P, Dash PK, Zhang W, Gendelman HE (2012). Biochemical and biologic characterization of exosomes and microvesicles as facilitators of HIV-1 infection in macrophages. J Immunol.

[20] Chertova E (2006). Proteomic and biochemical analysis of purified human immunodeficiency virus type 1 produced from infected monocyte-derived macrophages. J Virol.

[21] Aqil M, Mallik S, Bandyopadhyay S, Maulik U, Jameel S (2015). Transcriptomic Analysis of mRNAs in Human Monocytic Cells Expressing the HIV-1 Nef Protein and Their Exosomes. Biomed Res Int.

[22] Wiley RD, Gummuluru S (2006). Immature dendritic cell-derived exosomes can mediate HIV-1 trans infection. Proc Natl Acad Sci USA.

[23] Raymond AD (2011). HIV Type 1 Nef is released from infected cells in CD45(+) microvesicles and is present in the plasma of HIV-infected individuals. AIDS Res Hum Retroviruses.

[24] Fang Y (2007). Higher-order oligomerization targets plasma membrane proteins and HIV gag to exosomes. PLoS Biol.

[25] Narayanan A (2013). Exosomes derived from HIV-1-infected cells contain trans-activation response element RNA. The Journal of biological chemistry.

[26] Arenaccio C (2014). Exosomes from human immunodeficiency virus type 1 (HIV-1)-infected cells license quiescent CD4 + T lymphocytes to replicate HIV-1 through a Nef- and ADAM17-dependent mechanism. J Virol.

[27] Esser MT (2001). Differential incorporation of CD45, CD80 (B7-1), CD86 (B7-2), and major histocompatibility complex class I and II molecules into human immunodeficiency virus type 1 virions and microvesicles: implications for viral pathogenesis and immune regulation. J Virol.

[28] Khatua AK, Taylor HE, Hildreth JE, Popik W (2009). Exosomes packaging APOBEC3G confer human immunodeficiency virus resistance to recipient cells. J Virol.

[29] Li J (2013). Exosomes mediate the cell-to-cell transmission of IFN-alpha-induced antiviral activity. Nat Immunol.

[30] Eldh M (2010). Exosomes communicate protective messages during oxidative stress; possible role of exosomal shuttle RNA. PloS one.

[31] Saenz-Cuesta M (2015). Methods for extracellular vesicles isolation in a hospital setting. Front Immunol.

[32] Lobb RJ (2015). Optimized exosome isolation protocol for cell culture supernatant and human plasma. Journal of extracellular vesicles.

[33] Appay V, Sauce D (2008). Immune activation and inflammation in HIV-1 infection: causes and consequences. J Pathol.

[34] Sharma B (2014). Oxidative stress in HIV patients receiving antiretroviral therapy. Curr HIV Res.

[35] Hedlund M, Nagaeva O, Kargl D, Baranov V, Mincheva-Nilsson L (2011). Thermal- and oxidative stress causes enhanced release of NKG2D ligand-bearing immunosuppressive exosomes in leukemia/lymphoma T and B cells. PloS one.

[36] Cassol E (2013). Plasma metabolomics identifies lipid abnormalities linked to markers of inflammation, microbial translocation, and hepatic function in HIV patients receiving protease inhibitors. BMC Infect Dis.

[37] Cassol E (2015). Altered monomamine and acylcarnitine metabolites in HIV-positive and HIV-negative subjects with depression. J Acquir Immune Defic Syndr.

[38] Fuchs D (1990). Immune activation and decreased tryptophan in patients with HIV-1 infection. J Interferon Res.

[39] Fuchs D (1990). Decreased serum tryptophan in patients with HIV-1 infection correlates with increased serum neopterin and with neurologic/psychiatric symptoms. J Acquir Immune Defic Syndr.

[40] Huengsberg M (1998). Serum kynurenine-to-tryptophan ratio increases with progressive disease in HIV-infected patients. Clin Chem.

[41] Jenabian, M. A. *et al*. Immunosuppressive Tryptophan Catabolism and Gut Mucosal Dysfunction Following Early HIV Infection. *J Infect Dis*. 10.1093/infdis/jiv037 (2015). 10.1093/infdis/jiv037 25616404

[42] Murray, M. F. Tryptophan depletion and HIV infection: a metabolic link to pathogenesis. *Lancet Infect Dis* **3**, 644–652, https://doi.org/S1473309903007734 (2003). 10.1016/s1473-3099(03)00773-4 14522263

[43] Seymour CW (2013). Metabolomics in pneumonia and sepsis: an analysis of the GenIMS cohort study. Intensive Care Med.

[44] Kowalczyk-Pachel D (2016). Cysteine Metabolism and Oxidative Processes in the Rat Liver and Kidney after Acute and Repeated Cocaine Treatment. PloS one.

[45] Palucka KA, Taquet N, Sanchez-Chapuis F, Gluckman JC (1998). Dendritic cells as the terminal stage of monocyte differentiation. J. Immunol.

[46] Momen-Heravi F, Bala S, Kodys K, Szabo G (2015). Exosomes derived from alcohol-treated hepatocytes horizontally transfer liver specific miRNA-122 and sensitize monocytes to LPS. Scientific reports.

[47] De Truchis P (2007). Reduction in triglyceride level with N-3 polyunsaturated fatty acids in HIV-infected patients taking potent antiretroviral therapy: a randomized prospective study. J Acquir Immune Defic Syndr.

[48] Kim SJ (2010). Omega-3 and omega-6 fatty acids suppress ER- and oxidative stress in cultured neurons and neuronal progenitor cells from mice lacking PPT1. Neurosci Lett.

[49] Ngondi JL, Oben J, Forkah DM, Etame LH, Mbanya D (2006). The effect of different combination therapies on oxidative stress markers in HIV infected patients in Cameroon. AIDS Res Ther.

[50] Xiao J (2016). Cardiac progenitor cell-derived exosomes prevent cardiomyocytes apoptosis through exosomal miR-21 by targeting PDCD4. Cell Death Dis.

[51] Izquierdo-Useros N (2009). Capture and transfer of HIV-1 particles by mature dendritic cells converges with the exosome-dissemination pathway. Blood.

[52] Pepys MB, Hirschfield GM (2003). C-reactive protein: a critical update. J Clin Invest.

[53] Habersberger J (2012). Circulating microparticles generate and transport monomeric C-reactive protein in patients with myocardial infarction. Cardiovasc Res.

[54] Noursadeghi M, Miller RF (2005). Clinical value of C-reactive protein measurements in HIV-positive patients. Int J STD AIDS.

[55] Lau B (2006). C-reactive protein is a marker for human immunodeficiency virus disease progression. Arch Intern Med.

[56] Mathivanan S (2010). Proteomics analysis of A33 immunoaffinity-purified exosomes released from the human colon tumor cell line LIM1215 reveals a tissue-specific protein signature. Mol Cell Proteomics.

[57] Demory Beckler M (2013). Proteomic analysis of exosomes from mutant KRAS colon cancer cells identifies intercellular transfer of mutant KRAS. Mol Cell Proteomics.

[58] He M (2015). Hepatocellular carcinoma-derived exosomes promote motility of immortalized hepatocyte through transfer of oncogenic proteins and RNAs. Carcinogenesis.

[59] Liang B (2013). Characterization and proteomic analysis of ovarian cancer-derived exosomes. J Proteomics.

[60] Cheng P, Gabrilovich D (2008). Notch signaling in differentiation and function of dendritic cells. Immunol Res.

[61] Cheng P, Zhou J, Gabrilovich D (2010). Regulation of dendritic cell differentiation and function by Notch and Wnt pathways. Immunol Rev.

[62] Barchet W, Cella M, Colonna M (2005). Plasmacytoid dendritic cells–virus experts of innate immunity. Semin Immunol.

[63] Perez-Cabezas B (2011). Ligation of Notch receptors in human conventional and plasmacytoid dendritic cells differentially regulates cytokine and chemokine secretion and modulates Th cell polarization. J Immunol.

[64] Grigorian A, Hurford R, Chao Y, Patrick C, Langford TD (2008). Alterations in the Notch4 pathway in cerebral endothelial cells by the HIV aspartyl protease inhibitor, nelfinavir. BMC Neurosci.

[65] Vujkovic-Cvijin I (2013). Dysbiosis of the gut microbiota is associated with HIV disease progression and tryptophan catabolism. Sci Transl Med.

[66] Douek DC, Picker LJ, Koup RA (2003). T cell dynamics in HIV-1 infection. Annu Rev Immunol.

[67] Shiroguchi K, Jia TZ, Sims PA, Xie XS (2012). Digital RNA sequencing minimizes sequence-dependent bias and amplification noise with optimized single-molecule barcodes. Proc Natl Acad Sci USA.

[68] Muller L, Mitsuhashi M, Simms P, Gooding WE, Whiteside TL (2016). Tumor-derived exosomes regulate expression of immune function-related genes in human T cell subsets. Scientific reports.

[69] Geyer PE (2016). Plasma Proteome Profiling to Assess Human Health and Disease. Cell Syst.

[70] Gan X (1998). Cocaine infusion increases interferon-gamma and decreases interleukin-10 in cocaine-dependent subjects. Clin Immunol Immunopathol.

[71] Parikh N (2014). Cocaine alters cytokine profiles in HIV-1-infected African American individuals in the DrexelMed HIV/AIDS genetic analysis cohort. J Acquir Immune Defic Syndr.

[72] Siegel AJ (2002). Effect of cocaine usage on C-reactive protein, von Willebrand factor, and fibrinogen. Am J Cardiol.

[73] Valente MJ, Carvalho F, Bastos M, de Pinho PG, Carvalho M (2012). Contribution of oxidative metabolism to cocaine-induced liver and kidney damage. Curr Med Chem.

[74] Walker, J. *et al*. Total antioxidant capacity is significantly lower in cocaine-dependent and methamphetamine-dependent patients relative to normal controls: results from a preliminary study. *Hum Psychopharmacol*, 10.1002/hup.2430 (2014). 10.1002/hup.2430 PMC4280317 25087849

[75] Rider MA, Hurwitz SN, Meckes DG (2016). ExtraPEG: A Polyethylene Glycol-Based Method for Enrichment of Extracellular Vesicles. Scientific reports.

[76] Baranyai T (2015). Isolation of Exosomes from Blood Plasma: Qualitative and Quantitative Comparison of Ultracentrifugation and Size Exclusion Chromatography Methods. Plos One.

[77] Marimpietri D (2013). Proteome profiling of neuroblastoma-derived exosomes reveal the expression of proteins potentially involved in tumor progression. Plos One.

[78] Evans AM, DeHaven CD, Barrett T, Mitchell M, Milgram E (2009). Integrated, nontargeted ultrahigh performance liquid chromatography/electrospray ionization tandem mass spectrometry platform for the identification and relative quantification of the small-molecule complement of biological systems. Anal Chem.

[79] Dehaven CD, Evans AM, Dai H, Lawton KA (2010). Organization of GC/MS and LC/MS metabolomics data into chemical libraries. Journal of cheminformatics.

[80] Goetzl EJ (2015). Altered lysosomal proteins in neural-derived plasma exosomes in preclinical Alzheimer disease. Neurology.

[81] Farrah T (2011). A high-confidence human plasma proteome reference set with estimated concentrations in PeptideAtlas. Mol Cell Proteomics.

[82] Nair, S., Archer, G. E. & Tedder, T. F. Isolation and generation of human dendritic cells. *Current protocols in immunolog*y Chapter 7, Unit732, 10.1002/0471142735.im0732s99 (2012). 10.1002/0471142735.im0732s99 PMC4559332 23129155

[83] Shelke, G. V., Lasser, C., Gho, Y. S. & Lotvall, J. Importance of exosome depletion protocols to eliminate functional and RNA-containing extracellular vesicles from fetal bovine serum. *Journal of extracellular vesicles* **3**, 10.3402/jev.v3.24783 (2014). 10.3402/jev.v3.24783 PMC4185091 25317276

[84] Geiss GK (2008). Direct multiplexed measurement of gene expression with color-coded probe pairs. Nat Biotechnol.

