# Supplementary material for: Exosome markers associated with immune activation and oxidative stress in HIV patients on antiretroviral therapy
Source: Sci Rep. 2018 May 8;8:7227. doi: 10.1038/s41598-018-25515-4 (PMC5940833; doi:10.1038/s41598-018-25515-4)
Supplement: Supplementary file 1 — Supplementary Information [file 41598_2018_25515_MOESM1_ESM.pdf]

Exosome markers associated with immune activation and oxidative stress in HIV patients on antiretroviral therapy

Sukrutha Chettimada[1], David R. Lorenz[1], Vikas Misra[1], Simon T. Dillon[2,3], Roger K. Reeves[4], Cordelia Manickam[4], Susan Morgello[5], Gregory D. Kirk[6], Shruti H. Mehta[6], and Dana Gabuzda[1,7]

Affiliations:

[1]Dana-Farber Cancer Institute, Department of Cancer Immunology and Virology, Boston, MA

[2] BIDMC Genomics, Proteomics, Bioinformatics and Systems Biology Center, Beth Israel Deaconess Medical Center, Boston, MA

[3]Department of Medicine, Beth Israel Deaconess Medical Center, Harvard Medical School, Boston, MA

[4]Center for Virology and Vaccine Research, Beth Israel Deaconess Medical Center, Boston, MA

[5]Department of Neurology, Neuroscience and Pathology, Mount Sinai Medical Center, New York, NY

[6]Department of Epidemiology, Bloomberg School of Public Health, Johns Hopkins University, Baltimore, MD

[7]Department of Neurology, Harvard Medical School, Boston MA

Corresponding author:

Dr. Dana Gabuzda, email: dana_gabuzda@dfci.harvard.edu

This file includes:

1. Supplementary Figure S1
2. Supplementary Figure S2
3. Supplementary Figure S3
4. Supplementary Figure S4
5. Supplementary Figure S5
6. Supplementary Figure S6
7. Supplementary Figure S7
8. Supplementary Table S6

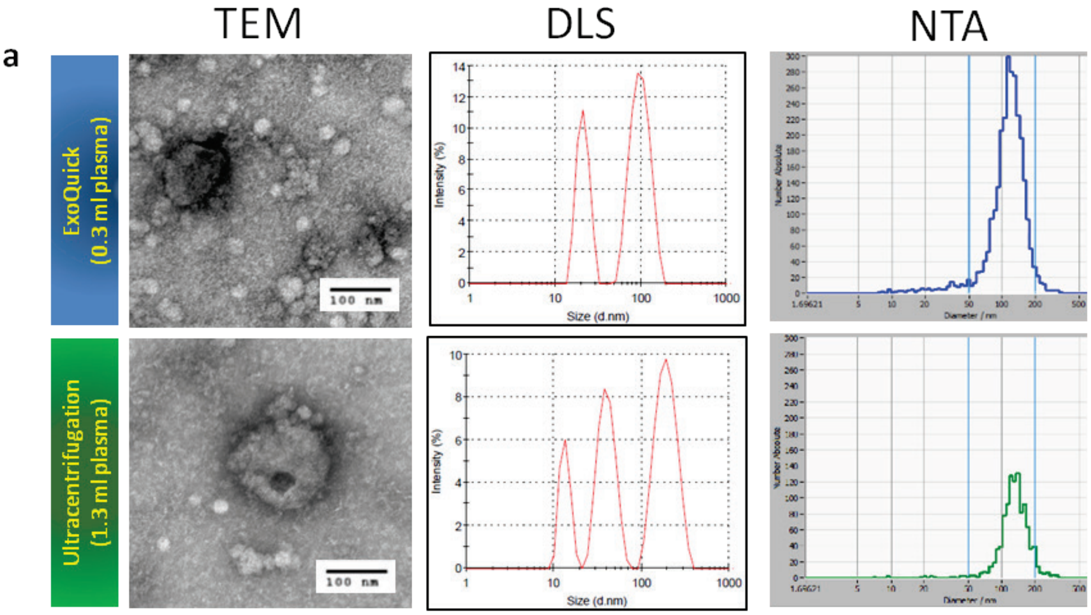

b

Lane	Pooled samples	EV conc. (particles/ ml plasma)
1	HIV-_ExoQk	1.13E+12
2	HIV+_ExoQk	1.58E+12
3	HIV-_UC	1.69E+10
4	HIV+_UC	3.00E+10

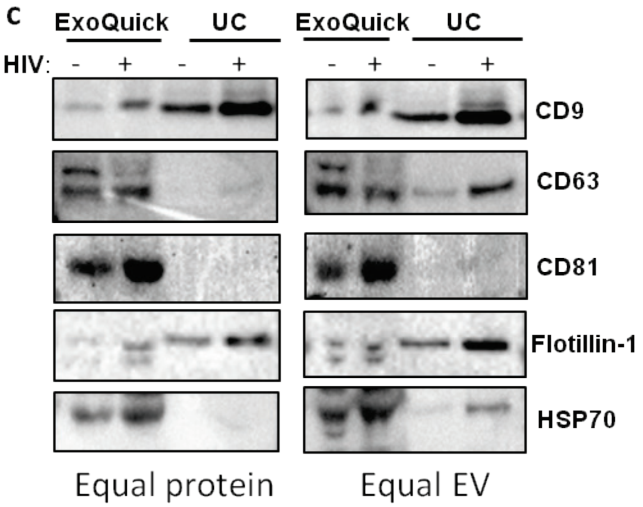

Supplementary Figure S1: Comparison of plasma exosomes isolation by ExoQuick reagent versus differential ultracentrifugation. 15 HIV+ or HIV- plasma samples were pooled to a total of 1.6 ml per sample. 0.3 ml plasma was used for ExoQuick method and 1.3 ml was used for differential ultracentrifugation (UC) method. **(a)** Exosomes were isolated from 0.3 ml plasma using ExoQuick reagent according to the manufacturer's protocol (upper panel), or from 1.3 ml plasma by UC (lower panel). Exosome morphology by TEM (first panel, scale bar=100 nm), size distribution by DLS (second panel), and EV concentration by and NTA (third panel) from HIV- sample is shown. **(b)** Table showing EV concentrations measured by NTA in HIV+ and HIV- samples. **(c)** Exosome fractions from the above experiments were run on SDS-PAGE gel by loading equal protein (left blot), or equal amounts of EV (right blot), between HIV- and HIV+ samples as measured by NTA (9.0E+10 and 1.2E+10 particles for ExoQuick and UC, respectively). Blots were probed for exosome marker proteins CD9, CD63, CD81, Flotillin-1, and HSP70 in pooled HIV- or HIV+ exosome fractions.

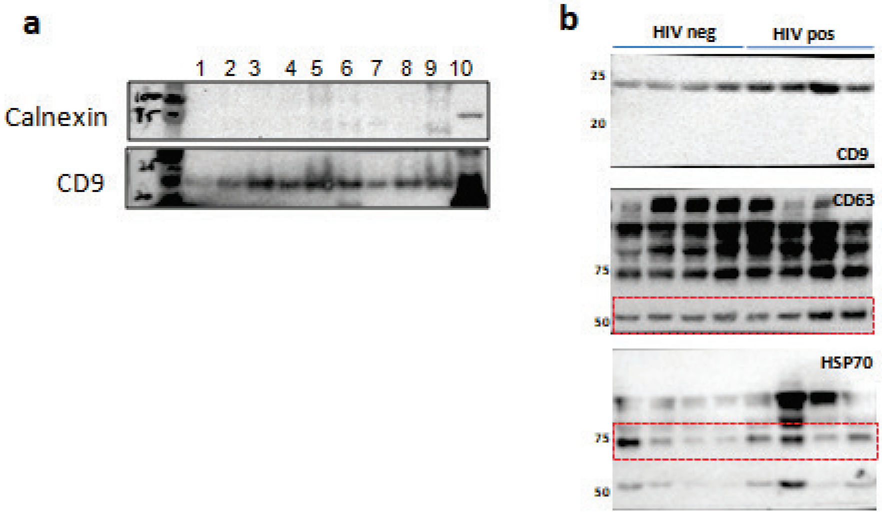

Supplementary Figure S2: (a) Exosome fractions are free of ER membrane contamination. Immunoblotting for ER protein, calnexin, and exosome marker, CD9, in plasma exosome fractions from HIV-negative (lanes 1-3), HIV-positive aviremic (lanes 4-6), HIV-positive viremic (lanes 7-9), and PBMC whole cell lysate (lane 10). **(b) Full length blots from Figure 2d.** Signal was developed by enhanced chemiluminescence and images were captured using a BioRad ChemiDoc™ Imaging System. Red dotted lines show cropping locations.

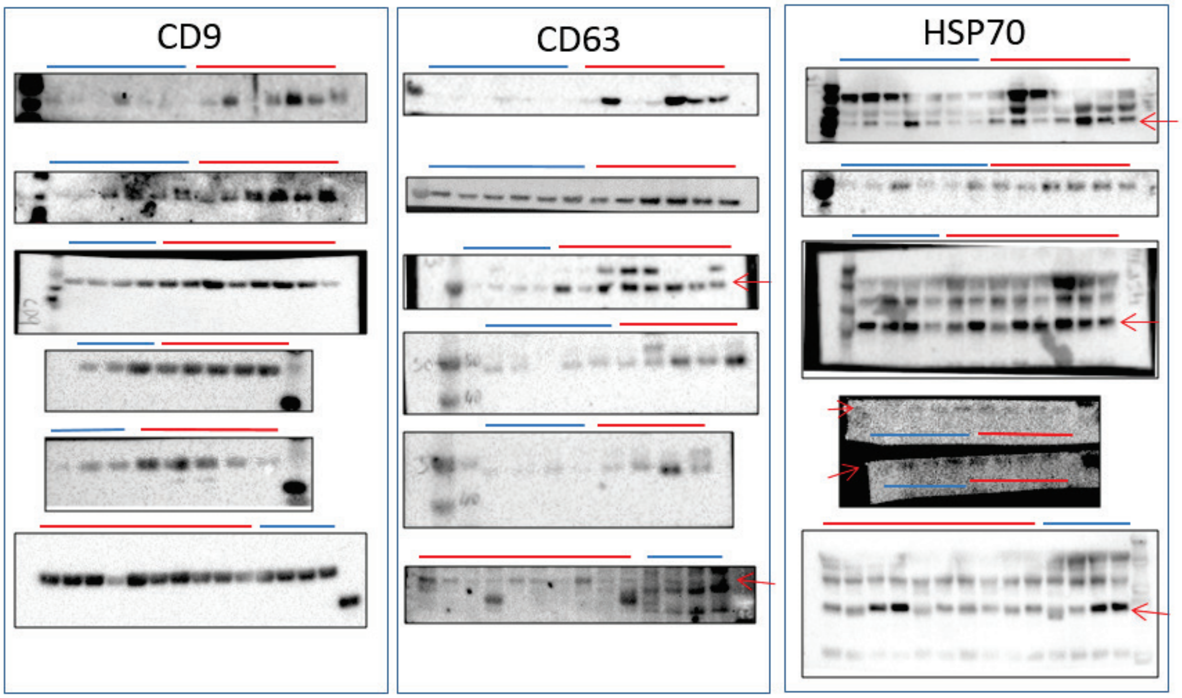

Supplementary Figure S3: Full length blots showing exosome markers in plasma exosome fractions from all subjects used in the study. Lanes under blue and red bars indicate HIV-negative and HIV-positive samples respectively. Signal was developed by enhanced chemiluminescence and images were captured using a BioRad ChemiDoc™ Imaging System.

a

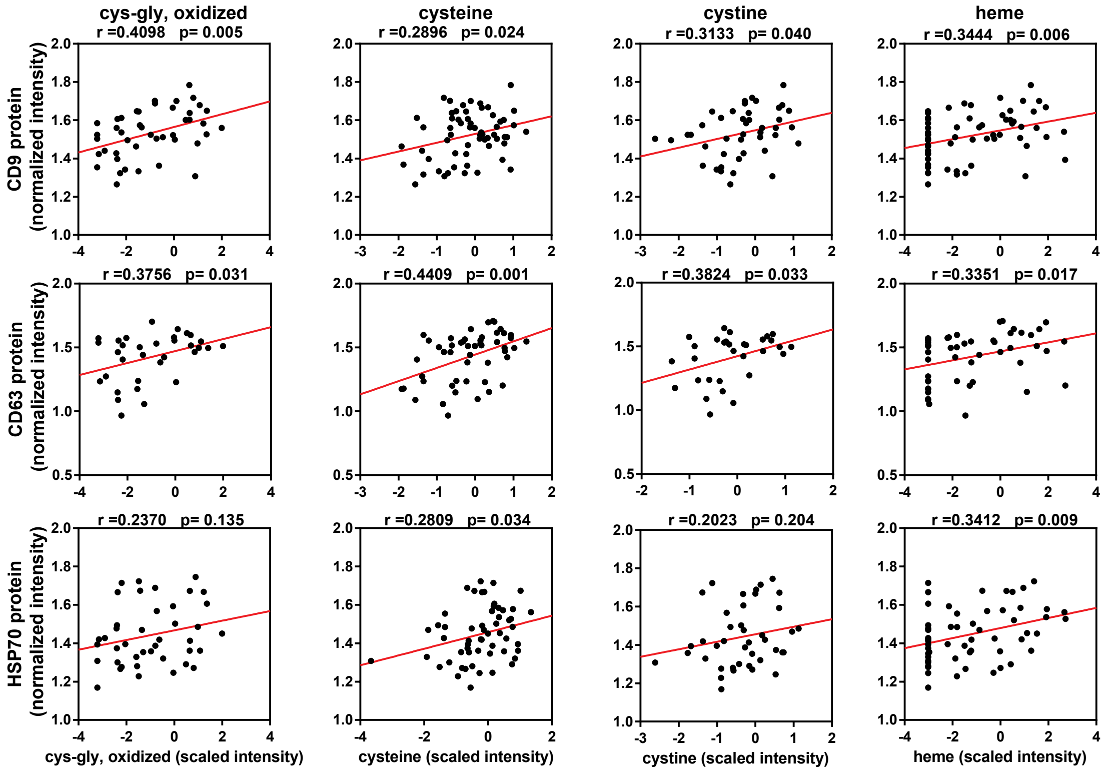

b

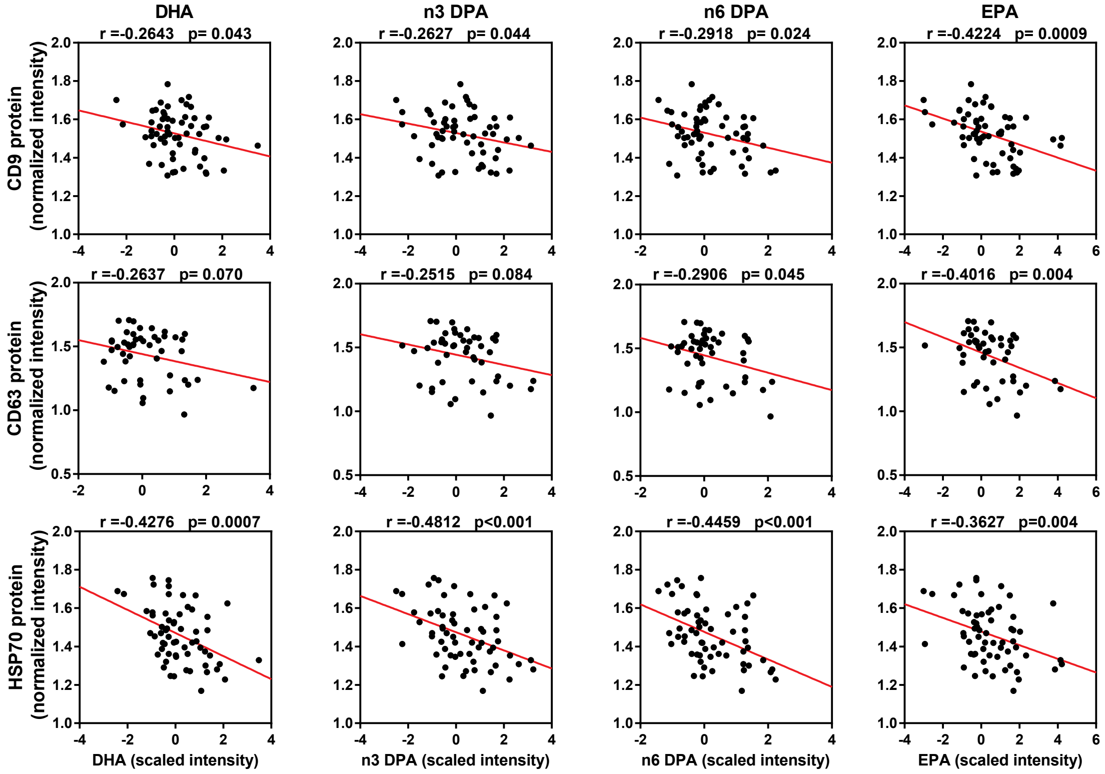

Supplementary Figure S4: Scatter plots showing relationships between oxidative stress metabolites (a), PUFA metabolites (b), and exosome markers band intensities (normalized to EV numbers)**.** Pearson correlation coefficient and p-value are shown above each plot. n= 63 subjects (24-36 HIV-positive, 20-26 HIV-negative). DHA, docosahexaenoate (22:6n3); n3 DPA, docosapentaenoate (22:5n3); n6 DPA, docosapentaenoate (22:5n6) and EPA, eicosapentaenoate (20:5n3)

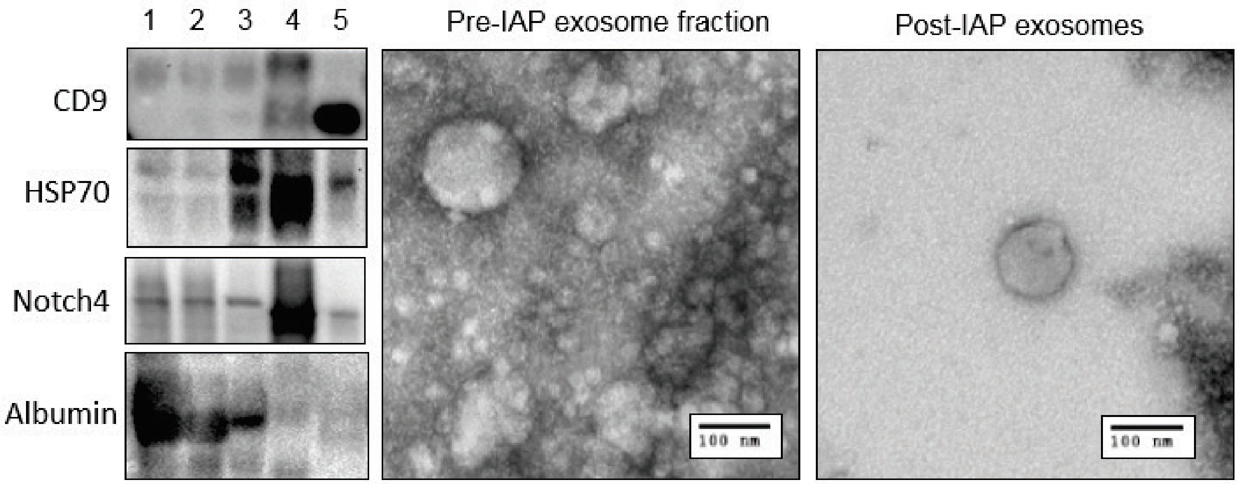

Supplementary Figure S5: Purification of exosome fraction by immunoaffinity purification. Left panel shows immunoblotting for exosome markers (CD9, HSP70), Notch4, and albumin in plasma (lane 1), albumin depleted-plasma (lane 2), exosome fraction (lane 3), pure exosomes from IAP (lane 4), and IgG control (lane 5). Right panel shows TEM image of exosomes in exosome fraction (left) and after exosome IAP (right).

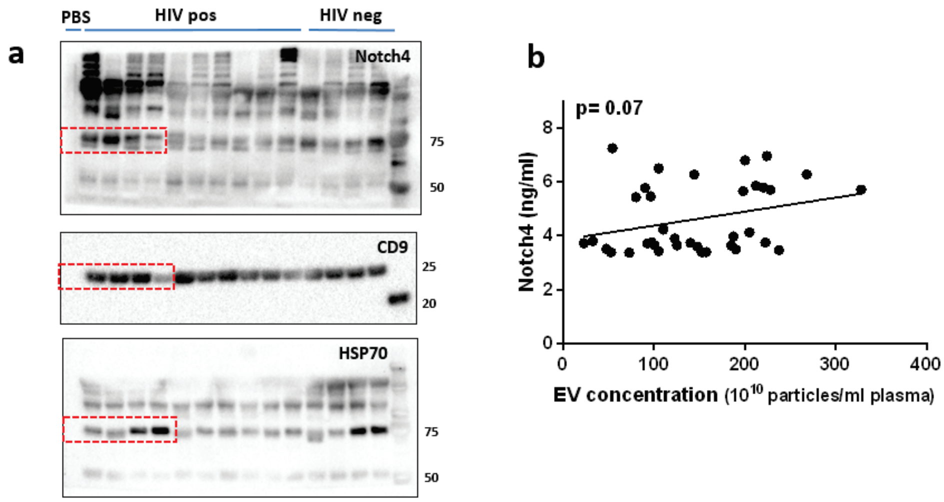

Supplementary Figure S6: (a) Full length blots from Figure 4. Signal was developed by enhanced chemiluminescence and images were captured using a BioRad ChemiDoc™ Imaging System. Red dotted lines show cropping locations. **(b) Scatter plot showing relationship between Notch4 levels and EV numbers.** Pearson **c**orrelation of Notch4 protein (as measured by ELISA) with EV numbers. n= 36 subjects (24 HIV-positive, 12 HIV-negative).

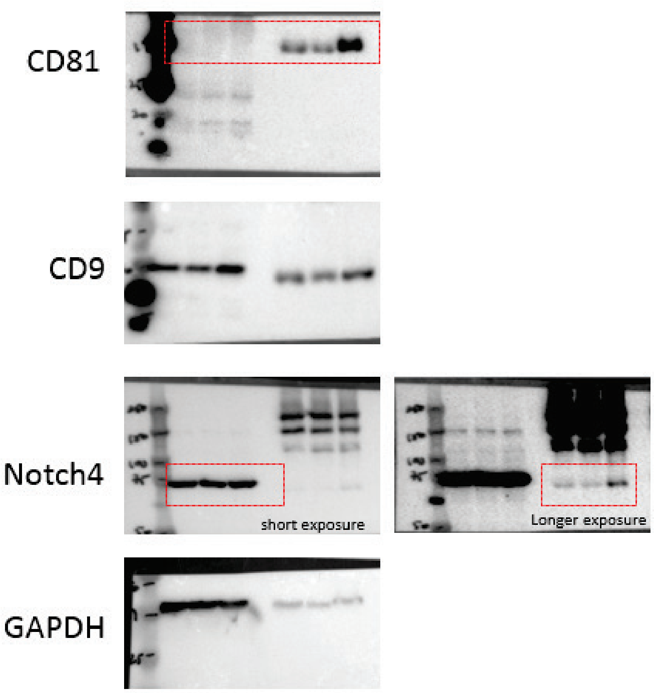

Supplementary Figure S7: Full length blots from Figure 6. Signal was developed by enhanced chemiluminescence and images were captured using a BioRad ChemiDoc™ Imaging System. Red dotted lines show cropping locations.

Supplementary Table S6. List of antibodies used in polychromatic flow cytometry

Antibody	Fluorochrome	Manufacturer	Clone
anti-BDCA-1 (CD1c)	APC	Miltenyi Biotech	AD5-8E7
anti-BDCA-2 (CD303)	FITC	Miltenyi Biotech	AC144
anti-CD11c	Alexa Fluor 700	BD Biosciences	S-HCL-3
anti-CD14	BV650	BD Biosciences	L-200
anti-CD16	BUV496	BD Biosciences	3G8
anti-CD19	BUV395	BD Biosciences	SJ25C1
anti-CD3	BV421	BD Biosciences	SP34-2
anti-CD4	PerCP-Cy5-5	BD Biosciences	L-200
anti-CD56	PE-Cy7	BD Biosciences	NCAM16.2
anti-CD8	APC-H7	BD Biosciences	SK1
anti-HLA-DR	ECD	Beckman-Coulter	Immu-357
anti-Notch4	PE	BD Biosciences	MHN4-2

Abbreviations: BDCA, Blood dendritic cell antigen; HLA-DR, human leukocyte antigen–DR
